# Association of Fetal Growth Restriction With Neurocognitive Function After Repeated Antenatal Betamethasone Treatment vs Placebo: Secondary Analysis of the ACTORDS Randomized Clinical Trial

**DOI:** 10.1001/jamanetworkopen.2018.7636

**Published:** 2019-02-01

**Authors:** Robert D. Cartwright, Caroline A. Crowther, Peter J. Anderson, Jane E. Harding, Lex W. Doyle, Christopher J. D. McKinlay

**Affiliations:** 1 Liggins Institute, University of Auckland, Auckland, New Zealand; 2 Discipline of Obstetrics and Gynaecology, School of Medicine, University of Adelaide, Adelaide, Australia; 3 Monash Institute of Cognitive and Clinical Neurosciences, Monash University, Melbourne, Australia; 4 Clinical Sciences, Murdoch Children’s Research Institute, Parkville, Australia; 5 Department of Paediatrics, University of Melbourne, Parkville, Australia; 6 Department of Obstetrics and Gynaecology, The Royal Women’s Hospital, University of Melbourne, Parkville, Australia; 7 Department of Paediatrics: Child and Youth Health, University of Auckland, Auckland, New Zealand; 8 Kidz First Neonatal Care, Counties Manukau Health, Auckland, New Zealand

## Abstract

**Importance:**

Repeated doses of antenatal betamethasone are recommended for women at less than 32 weeks’ gestation with ongoing risk of preterm birth. However, concern that this therapy may be associated with adverse neurocognitive effects in children with fetal growth restriction (FGR) remains.

**Objective:**

To determine the influence of FGR on the effects of repeated doses of antenatal betamethasone on neurocognitive function in midchildhood.

**Design, Setting, and Participants:**

This preplanned secondary analysis of data from the multicenter Australasian Collaborative Trial of Repeat Doses of Corticosteroids (ACTORDS) included women at less than 32 weeks’ gestation with ongoing risk of preterm birth (<32 weeks) at least 7 days after an initial course of antenatal corticosteroids who were treated at 23 hospitals across Australia and New Zealand from April 1, 1998, through July 20, 2004. Participants were randomized to intramuscular betamethasone or saline placebo; treatment could be repeated weekly if the woman was judged to be at continued risk of preterm birth. All surviving children were invited to a midchildhood outcome study. Data for this study were collected from October 27, 2006, through March 18, 2011, and analyzed from June 1 through 30, 2018.

**Interventions:**

At 6 to 8 years of corrected age, children were assessed by a pediatrician and psychologist for neurosensory and cognitive function, and parents completed standardized questionnaires.

**Main Outcomes and Measures:**

The prespecified primary outcomes were survival free of any disability and death or survival with moderate to severe disability.

**Results:**

Of 1059 eligible children, 988 (55.0% male; mean [SD] age at follow-up, 7.5 [1.1] years) were assessed at midchildhood. The FGR rate was 139 of 493 children (28.2%) in the repeated betamethasone treatment group and 122 of 495 (24.6%) in the placebo group (*P* = .20). Primary outcome rates were similar between treatment groups for the FGR and non-FGR subgroups, with no evidence of an interaction effect for survival free of any disability (FGR group, 108 of 144 [75.0%] for repeated betamethasone treatment vs 91 of 126 [72.2%] for placebo groups [odds ratio [OR], 1.1; 95% CI, 0.6-1.9]; non-FGR group, 267 of 335 [79.7%] for repeated betamethasone vs 283 of 358 [79.0%] for placebo groups [OR, 1.0; 95% CI, 0.7-1.5]; *P* = .77) and death or moderate to severe disability (FGR group, 21 of 144 [14.6%] for repeated betamethasone treatment vs 20 of 126 [15.9%] for placebo groups [OR, 0.9; 95% CI, 0.4-1.9]; non-FGR group, 29 of 335 [8.6%] for repeated betamethasone vs 36 of 358 [10.0%] for placebo [OR, 0.8; 95% CI, 0.4-1.3]; *P* = .84).

**Conclusions and Relevance:**

In this study, repeated antenatal betamethasone treatment compared with placebo was not associated with adverse effects on neurocognitive function at 6 to 8 years of age, even in the presence of FGR. Physicians should use repeated doses of antenatal corticosteroids when indicated before preterm birth, regardless of FGR, in view of the associated neonatal benefits and absence of later adverse effects.

**Trial Registration:**

anzctr.org.au Identifier: ACTRN12606000318583

## Key Points

**Question:**

Does fetal growth restriction influence neurocognitive function in midchildhood after repeated antenatal betamethasone treatment?

**Findings:**

In the 988 children followed up at 6 to 8 years of age in this secondary analysis of a placebo-controlled randomized clinical trial, exposure to repeated antenatal betamethasone treatment was not associated with adverse effects on survival free of any disability, death, or survival with moderate to severe disability, even in the presence of fetal growth restriction.

**Meaning:**

Health care professionals should use repeated doses of antenatal corticosteroids when indicated before preterm birth, regardless of fetal growth restriction, in view of the associated neonatal benefits and absence of later adverse effects.

## Teaser

This secondary analysis of a randomized clinical trial evaluates whether fetal growth restriction is associated with the effects of repeated doses of antenatal betamethasone on neurocognitive function in a cohort of children followed up in midchildhood.

## Introduction

Antenatal corticosteroid therapy remains one of the most effective treatments for preterm infants, and administration of a repeated dose or doses in women who are at ongoing risk of preterm birth at least 7 days after an initial course results in additional neonatal benefits.^1^ These benefits include reduced risk of preterm lung disease (especially severe disease), other combined serious neonatal morbidity, and patent ductus arteriosus. The absolute benefits of repeated-dose therapy are similar to those of an initial course.^2^ These clinical data are supported by studies in animals and human fetal lung explants showing that optimal structural and functional maturation requires serial exposure of fetal tissues to corticosteroids.^3^

However, animal studies have also revealed the potential for adverse long-term effects on organ development with increasing fetal exposure to corticosteroids. This potential is of particular concern for neural tissues with demonstration in different species that repeated or higher doses of corticosteroids can result in reduced brain mass,^4^ compromised structural development and neuronal maturation,^5^ diminished cellular proliferation and differentiation,^6^ reduced population of hippocampal neurons,^7,8^ and adverse development of the hypothalamic-pituitary-adrenal axis.^9^ These findings have contributed to the cautious clinical recommendations on the use of repeated doses of antenatal corticosteroids.^10^

Recent evidence from the Australasian Collaborative Trial of Repeat Doses of Corticosteroids (ACTORDS) has shown that use of repeated doses of antenatal corticosteroids in humans is not associated with adverse effects in offspring at midchildhood, including neurocognitive function, learning, behavior, growth, lung function, and cardiometabolic function.^11,12^ Nevertheless, clinical uptake of repeated doses of corticosteroids has been limited, and concern remains about the safety of this therapy in the context of fetal growth restriction (FGR), which is commonly associated with very preterm birth.^13,14^ Preterm-born children with FGR are at increased risk of adverse long-term neurodevelopmental outcomes and behavioral dysfunction,^15^ but at present, no published data are available from randomized clinical trials on the efficacy and safety of repeated-dose corticosteroid therapy in this important clinical subgroup. Therefore, we undertook a secondary analysis of data from the ACTORDS to determine the influence of FGR on the effects of repeated doses of antenatal betamethasone on neurocognitive function and behavior in midchildhood.^11^

## Methods

### ACTORDS Trial

ACTORDS was a placebo-controlled, randomized clinical trial of repeated antenatal betamethasone treatment conducted at 23 collaborating hospitals across Australia and New Zealand.^2^ The full trial protocol appears in Supplement 1. Eligible women had a single, twin, or triplet pregnancy at less than 32 weeks’ gestation, with an ongoing risk of preterm birth at least 7 days after an initial course of antenatal corticosteroids. A total of 982 women (1146 fetuses) were randomized, via a central telephone service, to an intramuscular dose of betamethasone (Celestone Chronodose, consisting of 7.8 mg of betamethasone sodium phosphate and 6 mg of betamethasone acetate) or saline placebo. The treatment could be repeated each week if the woman was judged to be at continued risk of preterm birth, until 32 weeks’ gestation.^2^ At 2 years of corrected age, neurodevelopment, growth, and general health were similar between groups.^16^ Although the midchildhood assessment was not part of the original trial protocol, this assessment was planned before the completion of the 2-year follow-up owing to concerns about the potential for long-term adverse effects of fetal corticosteroid exposure.^11^ Written informed consent was obtained from caregivers, and children provided assent for assessment. The Midchildhood Outcomes Study was approved by the National Health and Disability Ethics Committee in New Zealand and by regional Health Research Ethics Committees in Australia. This report has been prepared according to the Consolidated Standards of Reporting Trials (CONSORT) reporting guidelines for clinical trials.

### Midchildhood Outcomes Study

All surviving children of mothers who had participated in ACTORDS were invited to partake in the Midchildhood Outcomes Study of neurocognitive function and general health at 6 to 8 years of corrected age.^11^ Children were assessed by a pediatrician and a psychologist who were blinded to treatment allocation.^11^ The pediatric assessment included a physical and neurologic examination, vision and hearing screening, and tests of fine and gross motor function using the Movement Assessment Battery for Children, Second Edition (MABC-2).^17^ Several children underwent assessment using the earlier edition of the MABC. Cerebral palsy was defined as a nonprogressive loss of motor function with disordered muscle tone or tendon reflexes^18^ and was graded according to gross motor function criteria of Palisano et al^19^ (mild, grade 1; moderate, grades 2-3; and severe, grades 4-5). Blindness consisted of visual acuity of worse than 20/200 in the better eye. Deafness consisted of hearing loss requiring hearing aids or worse.

The psychological assessment included the Wechsler Abbreviated Scale of Intelligence.^20^ The full-scale IQ was derived from the Vocabulary, Similarities, Block Design, and Matrix Reasoning subtests. Scores were age standardized with a normative mean (SD) of 100 (15). Intellectual impairment was classified as mild (IQ of 1-2 SDs below the mean), moderate (IQ of >2 to 3 SDs below the mean), and severe (IQ of >3 SDs below the mean). Children with severe intellectual impairment who were unable to complete the Wechsler Abbreviated Scale of Intelligence were assigned an IQ score of 40.

Attention was assessed using subtests from the Test of Everyday Attention for Children.^21^ Selective visual attention was assessed using the Sky Search subtest; sustained attention, the Score! subtest; shifting attention, the Creature Counting subtest; and divided attention, the Sky Search Dual Task subtest. Scores in the Sky Search, Score!, and Creature Counting subtests were age standardized (test mean [SD], 10 [3]). Performance in the Sky Search Dual Task subtest was determined by the mean of the proportion of visual targets correctly identified plus the proportion of correct auditory counting games multiplied by 100.^21^ The range of possible values is 0 to 100, and although this scoring procedure has no published norms, the mean (SD) score in a study of 173 control children at 8 years of age was 80.3 (16.5).^22^

Executive function was assessed using the Rey Complex Figure Test^23^ and the Fruit Stroop Task.^24^ The Complex Figure Test assesses complex spatial organization; children’s copying of a complex geometrical figure was scored for accuracy (maximum score of 36)^23^ and strategic organization.^25^ The Fruit Stroop Task assessed impulse control, with performance determined by the number of correct responses in 45 seconds (naming the true color of fruit that was presented in conflicting colors).^11^ Academic skills were assessed using the word reading, spelling, and math computation subtests of the Wide Range Achievement Test, fourth edition.^26^ Each scale is age standardized with a normative mean (SD) of 100 (15).

Caregivers completed questionnaires, including the Strengths and Difficulties Questionnaire to assess general behavioral and emotional problems,^27^ the Behavior Rating Inventory of Executive Function to assess behavioral manifestations of inattention and executive function,^28^ and the Conners’ ADHD/*DSM-IV* Scales^29^ to assess for features of attention-deficit/hyperactivity disorder. Neurosensory disability included cerebral palsy, intellectual impairment, or blindness or deafness and was graded as mild (mild cerebral palsy or IQ of 70-84), moderate (deafness, moderate cerebral palsy, or IQ of 55-69), or severe (blindness, severe cerebral palsy, or IQ of <55).

### Study Hypothesis

Children who completed 1 or more of the neurocognitive tests at 6 to 8 years of corrected age were included in this secondary analysis of data from the midchildhood assessments of the ACTORDS.^11^ The prespecified primary outcomes for this study were survival free of any neurosensory disability and death or moderate to severe disability. To reduce the risk of type I error, the following secondary outcomes were selected a priori as key indicators of function in the each neurocognitive domain: (1) cognition using full-scale IQ and cognitive impairment (IQ <85); (2) motor using cerebral palsy and low motor function (MABC total score <15th centile); (3) attention using Test of Everyday Attention for Children subtest scores; (4) executive function using Rey Complex Figure Test accuracy and organization scores and the number of correct Fruit Stroop Task responses (trial 4); (5) educational achievement using Wide Range Achievement Test, edition 4, scores in reading, spelling, and mathematics; and (6) behavior using the Strengths and Difficulties Questionnaire Total Difficulties score (range, 0-40, with 14-16 indicating borderline and ≥17 abnormal),^27^ Behavior Rating Inventory of Executive Function Global Executive Composite t score (mean [SD], 50 [10]), and Conners’ ADHD/*DSM-IV* Scales ADHD Index t score (mean [SD], 50 [10]).

We hypothesized that exposure to repeated antenatal betamethasone treatment, compared with a single course of treatment, would have adverse effects on neurosensory function, general cognition, attention, executive function, academic performance, and behavior at 6 to 8 years of corrected age in children with FGR but not for those with normal prenatal growth. As previously described,^30^ FGR was defined a priori as 1 or more of the following: obstetric diagnosis of FGR at trial entry; cesarean delivery for FGR; or customized birth weight of no greater than the third centile (GROW, version 6.7.8.3; Perinatal Institute). Although this definition includes postrandomization factors, these were judged to be important because antenatal diagnosis of FGR substantially underrepresents the true incidence of FGR in the preterm population.^14^ However, we used a conservative birth weight threshold of the third centile. Customized centiles, which incorporate fetal growth curves and account for normal maternal constraint on fetal growth, were used because these have been shown to improve detection of FGR.^31^ Further, meta-analysis of randomized clinical trials has shown that repeated doses of corticosteroids do not increase the risk of being small for gestational age.^1^

### Statistical Analysis

Analyses were performed using SAS software (version 9.4; SAS Institute, Inc). Data are presented as number (percentage) or mean (SD). For all prespecified outcomes, treatment groups were compared using generalized linear models with adjustment for gestational age at trial entry, preterm prelabor rupture of membranes, antepartum hemorrhage, country of birth, and clustering of children from multiple pregnancy by generalized estimating equations.^2^ The influence of FGR on treatment effect was assessed by an interaction test. Treatment effects within the FGR and non-FGR subgroups are reported as odds ratios (ORs) for binary outcomes or mean difference (MD) for continuous outcomes with 95% CI. Two-tailed α < .05 was considered statistically significant.

## Results

Of the 1059 surviving children eligible for the Midchildhood Outcomes Study, 988 (445 [45.0%] female and 543 [55.0%] male; mean [SD] age at follow-up, 7.5 [1.1] years) completed 1 or more tests of neurocognitive function (repeated betamethasone treatment, 493 participants; placebo, 495 participants) (Figure).^11^ The rate of FGR was similar between those exposed to repeated betamethasone therapy (139 of 493 [28.2%]) and placebo (122 of 495 [24.6%]) (*P* = .20).

**Figure.**
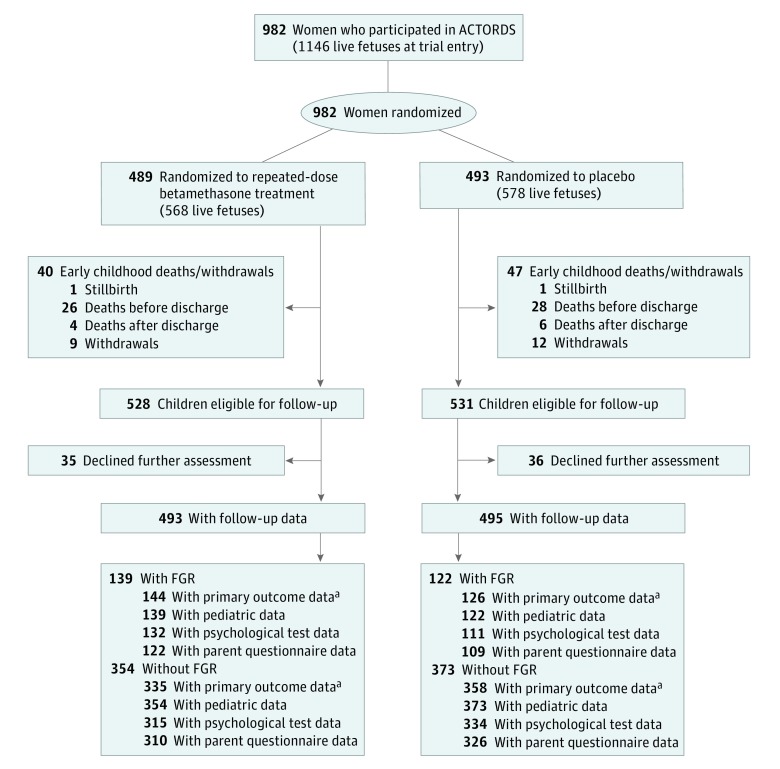
CONSORT Diagram of Participant Randomization, Treatment, and Follow-up for Neurodevelopment at Midchildhood ACTORDS indicates Australasian Collaborative Trial of Repeat Doses of Corticosteroids; FGR, fetal growth restriction. ^a^Denominator for primary outcome includes postrandomization deaths.

The FGR subgroup, compared with the non-FGR subgroup, was characterized by older mean maternal age (31.9 [5.8] vs 30.4 [5.9] years; *P* = .002), higher maternal parity (parity ≥4, 29 of 216 [13.4%] vs 54 of 673 [8.0%]; *P* = .02), and increased rates of multiple pregnancy (79 of 216 [36.6%] vs 93 of 673 [13.8%]; *P* < .001) and preeclampsia (67 of 216 [31.0%] vs 27 of 673 [4.0%]; *P* < .001) (Table 1). Fetal growth restriction was associated with lower rates of preterm prelabor rupture of membranes (35 of 216 [16.2%] vs 248 of 673 [36.8%]; *P* < .001), cervical incompetence (11 of 216 [5.1%] vs 67 of 673 [10.0%]; *P* = .03), antepartum hemorrhage (34 of 216 [15.7%] vs 218 of 673 [32.4%]; *P* < .001), and shorter mean gestation (31.8 [3.1] vs 32.9 [4.0] weeks; *P* < .001) (Table 1). Women in the FGR group were less likely to speak English at home (177 of /216 [81.9%] vs 594 of 673 [88.3%]; *P* = .02) (Table 1). Neonates with FGR had substantially reduced *z* scores for mean birth weight (−1.2 [0.8] vs 0.1 [0.7]; *P* < .001) and head circumference (−0.9 [0.9] vs 0.2 [1.1]; *P* < .001) and increased rates of mechanical ventilation (154 of 261 [59.0%] vs 344 of 727 [47.3%]; *P* = .002); and serious neonatal morbidity (78 of 261 [29.9%] vs 132 of 727 [18.2%]; *P* < .001) (Table 1).

**Table 1.** Characteristics of Children and Their Mothers at Midchildhood Assessment

Characteristic	FGR Group			Non-FGR Group		
	Total	Repeated-Dose Betamethasone Treatment	Placebo	Total	Repeated-Dose Betamethasone Treatment	Placebo
**Maternal Characteristics**						
No. of mothers	216	118	98	673	330	343
Age, mean (SD), y	31.9 (5.8)^a^	31.5 (6.0)	32.4 (5.4)	30.4 (5.9)	30.5 (5.9)	30.4 (5.9)
Parity, No. (%)						
0	77 (35.6)	43 (36.4)	34 (34.7)	207 (30.8)	102 (30.9)	105 (30.6)
1-3	110 (50.9)^a^	58 (49.2)	52 (53.1)	412 (61.2)	204 (61.8)	208 (60.6)
≥4	29 (13.4)^a^	17 (14.4)	12 (12.2)	54 (8.0)	24 (7.3)	30 (8.7)
Multiple pregnancy, No. (%)	79 (36.6)^a^	39 (33.1)	40 (40.8)	93 (13.8)	48 (14.5)	45 (13.1)
Smoking during pregnancy, No. (%)	58 (26.9)	36 (30.5)	22 (22.4)	210 (31.2)	97 (29.4)	113 (32.9)
Gestational age at trial entry, mean (SD), wk	28.5 (2.1)	28.5 (2.1)	28.5 (2.1)	28.3 (2.2)	28.3 (2.3)	28.4 (2.2)
Main reasons for risk of preterm birth, No. (%)^b^						
Preterm prelabor rupture of membranes, No. (%)	35 (16.2)^a^	19 (16.1)	16 (16.3)	248 (36.8)	108 (32.7)^c^	140 (40.8)
Preterm labor	34 (15.7)^a^	23 (19.5)	11 (11.2)	192 (28.5)	98 (29.7)	94 (27.4)
Severe FGR	46 (21.3)^a^	26 (22.0)	20 (20.4)	NA	NA	NA
Preeclampsia	67 (31.0)^a^	32 (27.1)	35 (35.7)	27 (4.0)	16 (4.8)	11 (3.2)
Cervical incompetence	11 (5.1)^a^	5 (4.2)	6 (6.1)	67 (10.0)	38 (11.5)	29 (8.4)
Antepartum hemorrhage	34 (15.7)^a^	20 (16.9)	14 (14.3)	218 (32.4)	118 (35.8)	100 (29.2)
Multiple pregnancy	13 (6.0)	6 (5.1)	7 (7.1)	21 (3.1)	14 (4.2)	7 (2.0)
Other	63 (29.2)^a^	34 (28.8)	29 (29.6)	96 (14.3)	44 (13.3)	52 (15.2)
No. of trial treatments, No. (%)						
1	92 (42.6)	47 (39.8)	45 (45.9)	274 (40.7)	130 (39.4)	144 (42.0)
2-3	72 (33.3)	33 (28.0)	39 (39.8)	245 (36.4)	118 (35.8)	127 (37.0)
≥4	52 (24.1)	38 (32.2)^c^	14 (14.3)	154 (22.9)	82 (24.8)	72 (21.0)
Speak only English at home, No. (%)	177 (81.9)^a^	96 (81.4)	81 (82.6)	594 (88.3)	293 (88.8)	301 (87.8)
Intact family, No. (%)	146 (67.6)	80 (67.8)	66 (67.3)	413 (61.4)	196 (59.4)	217 (63.3)
Occupation home duties only, No. (%)	66 (30.6)	41 (34.7)	25 (25.5)	186 (27.6)	87 (26.4)	99 (28.9)
**Neonatal Characteristics**						
No. of infants	261	139	122	727	354	373
Female, No. (%)	126 (48.3)	74 (53.2)	52 (42.6)	319 (43.9)	144 (40.7)	175 (46.9)
Gestational age at birth, mean (SD), wk	31.8 (3.1)^a^	32.2 (3.2)^c^	31.2 (3.0)	32.9 (4.0)	32.9 (4.1)	33 (3.9)
Birth weight, mean (SD), g	1407 (525)^a^	1476 (519)^c^	1328 (521)	2095 (830)	2090 (867)	2100 (795)
Birth weight *z* score, mean (SD)	−1.2 (0.8)^a^	−1.3 (0.8)	−1.2 (0.8)	0.1 (0.7)	0.1 (0.8)	0.2 (0.7)
Head circumference *z* score, mean (SD)	−0.9 (0.9)^a^	−0.9 (1.0)	−0.9 (0.9)	0.2 (1.1)	0.1 (1.1)	0.2 (1.0)
Respiratory distress syndrome, No. (%)^d^	106 (40.6)	43 (30.9)^c^	63 (51.6)	245 (33.7)	107 (30.2)	138 (37.0)
Severity of neonatal lung disease, No. (%)^e^		^c^			^c^	
Severe	40 (15.3)	11 (7.9)	29 (23.8)	85 (11.7)	29 (8.2)	56 (15.0)
Moderate	43 (16.5)	22 (15.8)	21 (17.2)	130 (17.9)	58 (16.4)	72 (19.3)
Mild	90 (34.5)	43 (30.9)	47 (38.5)	217 (29.8)	119 (33.6)	98 (26.3)
None	88 (33.7)	63 (45.3)	25 (20.5)	295 (40.6)	148 (41.8)	147 (39.4)
Mechanical ventilation, No. (%)	154 (59.0)^a^	67 (48.2)^c^	87 (71.3)	344 (47.3)	163 (46.0)	181 (48.5)
Oxygen therapy, No. (%)	159 (60.9)	73 (52.5)^c^	86 (70.5)	407 (56.0)	190 (53.7)	217 (58.2)
Surfactant, No. (%)	76 (29.1)	26 (18.7)^c^	50 (41.0)	182 (25.0)	81 (22.9)	101 (27.1)
Serious neonatal morbidity, No. (%)^f^	78 (29.9)^a^	31 (22.3)^c^	47 (38.5)	132 (18.2)	57 (16.1)	75 (20.1)

Abbreviations: FGR, fetal growth restriction; NA, not applicable.

^a^ *P* < .05 for comparison between subgroups (Fisher exact test or unpaired, 2-tailed *t* test).

^b^ Indicates at trial entry; categories are not mutually exclusive.

^c^ *P* < .05 for comparison between trial intervention groups within subgroup (Fisher exact test or *t* test).

^d^ Indicates clinical signs of respiratory distress syndrome and a ground-glass appearance on chest radiograph.

^e^ Mild indicates mean airway pressure (MAP) of less than 7 cm or fractional inspired oxygen (Fio_2_) of less than 0.40; moderate, MAP of 7 to less than 10 cm H_2_O or Fio_2_ 0.40 to 0.79; and severe, MAP of at least 10 cm or Fio_2_ of at least 0.80.

^f^ Indicates air leak syndrome, patent ductus arteriosus, need for oxygen therapy at 36 weeks’ postmenstrual age, severe intraventricular hemorrhage (grade 3 or 4), periventricular leukomalacia, proven necrotizing enterocolitis, and/or retinopathy of prematurity.

In the FGR subgroup, those exposed to repeated-dose betamethasone therapy were more likely than those exposed to placebo to have received at least 4 trial treatments (38 of 118 [32.2%] vs 14 of 98 [14.3%]; *P* = .002) and to be born at a later mean gestational age (32.2 [3.2] vs 31.2 [3.0] weeks; *P* ≤ .001). Repeated betamethasone therapy reduced the incidence of respiratory distress syndrome, the severity of neonatal lung disease, and serious neonatal morbidity, as well as the need for mechanical ventilation, oxygen, and surfactant therapy (Table 1).

In the non-FGR subgroup, those exposed to repeated betamethasone treatment were less likely than those exposed to placebo to have preterm prelabor rupture of membranes (108 of 330 [32.7%] vs 140 of 343 [40.8%]; *P* = .03) as the main reason for being at risk of preterm birth. Repeated betamethasone therapy significantly reduced the severity of neonatal lung disease (Table 1).

For the primary outcomes at 6 to 8 years of corrected age, rates were similar between treatment groups in the FGR and non-FGR subgroups, with no evidence of an interaction effect for survival free of any disability (FGR, 108 of 144 [75.0%] with repeated betamethasone vs 91 of 126 [72.2%] with placebo [odds ratio (OR), 1.1; 95% CI, 0.6-1.9]; non-FGR, 267 of 335 [79.7%] with repeated betamethasone vs 283 of 358 [79.0%] with placebo [OR, 1.0; 95% CI, 0.7-1.5]; *P* = .77) or for death or moderate to severe disability (FGR, 21 of 144 [14.6%] with repeated betamethasone vs 20 of 126 [15.9%] with placebo [OR, 0.9; 95% CI, 0.4-1.9]; non-FGR, 29 of 335 [8.6%] with repeated betamethasone vs 36 of 358 [10.0%] with placebo [OR, 0.8; 95% CI, 0.4-1.3]; *P* = .84) (Table 2).

**Table 2.** Neurocognitive Function at Midchildhood of Children Exposed to Repeated-Dose Betamethasone Treatment or Placebo

Outcome	Subgroup	Repeated-Dose Betamethasone Treatment Group		Placebo Group		Treatment Effect (95% CI)^a^	*P* Value for Interaction
		Data	Total No.	Data	Total No.		
Survival free of any disability, No. (%)^b^	FGR	108 (75.0)	144	91 (72.2)	126	OR, 1.1 (0.6 to 1.9)	.77
	Non-FGR	267 (79.7)	335	283 (79.0)	358	OR, 1.0 (0.7 to 1.5)	
Death or moderate to severe disability, No. (%)^b^	FGR	21 (14.6)	144	20 (15.9)	126	OR, 0.9 (0.4 to 1.9)	.84
	Non-FGR	29 (8.6)	335	36 (10.0)	358	OR, 0.8 (0.4 to 1.3)	
Cognition							
Full-scale IQ, mean (SD)	FGR	97.3 (16.1)	130	97.5 (14.3)	111	MD, −0.3 (−4.5 to 3.9)	.83
	Non-FGR	101.0 (16.1)	314	100.5 (16.2)	334	MD, 0.3 (−2.2 to 2.9)	
Full-scale IQ <85, No. (%)	FGR	20 (15.4)	130	15 (13.5)	111	OR, 1.1 (0.5 to 2.5)	.85
	Non-FGR	40 (12.7)	314	41 (12.3)	334	OR, 1.0 (0.6 to 1.7)	
Motor, No. (%)							
Cerebral palsy	FGR	6 (4.3)	139	5 (4.1)	122	OR, 1.0 (0.3 to 3.4)	.86
	Non-FGR	13 (3.7)	354	15 (4.1)	372	OR, 0.9 (0.4 to 1.9)	
Low movement, ABC total score <15th centile	FGR	43 (33.3)	129	36 (33.3)	108	OR, 0.9 (0.5 to 1.7)	.86
	Non-FGR	66 (21.8)	303	78 (23.9)	327	OR, 0.9 (0.6 to 1.4)	
TEA-Ch subtest score, mean (SD)^c^							
Selective attention: Sky Search	FGR	9.3 (3.1)	125	8.6 (3.4)	108	MD, 0.7 (−0.1 to 1.5)	.05
	Non-FGR	9.1 (3.1)	301	9.3 (3.2)	320	MD, −0.2 (−0.7 to 0.2)	
Sustained attention: Score!	FGR	8.7 (3.7)	123	9.0 (3.6)	103	MD, −0.2 (−1.2 to 0.7)	.68
	Non-FGR	8.7 (3.4)	299	8.6 (3.6)	305	MD, 0.0 (−0.5 to 0.6)	
Shifting attention: Creature Counting	FGR	9.1 (3.6)	104	8.8 (3.7)	90	MD, 0.3 (−0.7 to 1.3)	.83
	Non-FGR	9.8 (3.6)	273	9.6 (3.5)	280	MD, 0.1 (−0.4 to 0.7)	
Divided attention: Sky Search Dual Task	FGR	59.9 (26.9)	118	52.6 (30.2)	98	MD, 7.1 (−0.8 to 15.2)	.02
	Non-FGR	58.4 (29.8)	290	62.2 (28.7)	292	MD, −3.5 (−8.4 to 1.3)	
Executive function, mean (SD)							
Rey Complex Figure Accuracy score^d^	FGR	15.1 (7.1)	124	13.4 (7.4)	106	MD, 1.8 (−0.1 to 3.8)	.08
	Non-FGR	15.6 (7.9)	302	15.9 (7.9)	316	MD, −0.4 (−1.7 to 0.8)	
Rey Complex Figure Organization score^e^	FGR	3.6 (1.2)	124	3.4 (1.1)	106	MD, 0.3 (0.0 to 0.6)	.14
	Non-FGR	3.7 (1.1)	300	3.6 (1.1)	315	MD, 0.0 (−0.2 to 0.1)	
Fruit Stroop Task, No. correct (trial 4)	FGR	20.0 (8.4)	119	19.2 (7.7)	106	MD, 1.0 (−1.1 to 3.1)	.02
	Non-FGR	19.3 (8.2)^e^	294	21.3 (8.6)	310	MD, −2.1 (−3.5 to −0.8)	

Abbreviations: ABC, Assessment Battery for Children; FGR, fetal growth restriction; MD, mean difference; OR, odds ratio; TEA-Ch, Test of Everyday Attention for Children.

^a^ Adjusted for potential confounders (gestational age at trial entry, antepartum hemorrhage, preterm prelabor rupture of membranes, and country of birth) and clustering of children from multiple pregnancy.

^b^ Disability defined as any of cerebral palsy, blindness or deafness, or IQ of less than 85; moderate or severe, deafness, moderate to severe cerebral palsy, or IQ of less than 70.

^c^ Scores range from 0 to 20, with higher scores indicating better attention.

^d^ Scores range from 0 to 36, with higher scores indicating better executive function.

^e^ *P* < .05 for comparison between trial intervention groups within subgroup.

For the secondary outcomes of Sky Search Dual Task (divided attention) and Fruit Stroop Task (executive function), a significant interaction occurred for the effect of repeated antenatal betamethasone therapy and FGR. In the FGR subgroup, children exposed to repeated betamethasone performed better on the Sky Search Dual Task than those exposed to placebo; no significant difference was seen between treatment groups in the non-FGR subgroup (FGR MD, 7.1 [95% CI, −0.8 to 15.2]; non-FGR MD, −3.5 [95% CI, −8.4 to 1.3]; *P* = .02 for interaction) (Table 2). Conversely, in the non-FGR subgroup, children exposed to repeated betamethasone performed worse on the Fruit Stroop Task than those exposed to placebo; no significant difference was seen between treatment groups in the FGR subgroup for number correct (FGR MD, 1.0 [95% CI, −1.1 to 3.1]; non-FGR MD, −2.1 [95% CI, −3.5 to −0.8]; *P* = .02 for interaction) (Table 2). In post hoc analyses, these interactions remained significant after adjustment for maternal parity and number of trial treatments. For all other secondary outcomes, rates and scores were similar between the FGR and non-FGR subgroups, with no evidence of an interaction (Table 2, Table 3, and Table 4).

**Table 3.** Academic Skills at Midchildhood of Children Exposed to Repeated-Dose Betamethasone Treatment or Placebo

WRAT-4 Outcome^a^	Subgroup	Repeated-Dose Betamethasone Treatment Group		Placebo Group		Treatment Effect, MD (95% CI)^b^	*P* Value for Interaction
		Mean (SD) Score	Total No.	Mean (SD) Score	Total No.		
Reading	FGR	97.6 (14.7)	121	96.3 (17.9)	107	1.2 (−3.7 to 6.2)	.52
	Non-FGR	99.7 (17.2)	306	100.1 (17.7)	324	−0.4 (−3.2 to 2.4)	
Spelling	FGR	98.8 (14.0)	119	98.1 (16.5)	107	0.6 (−4.1 to 5.4)	.48
	Non-FGR	100.1 (16.0)	305	101.4 (16.9)	323	−1.2 (−4.0 to 1.4)	
Mathematics	FGR	94.7 (14.9)	120	95.2 (16.1)	107	−0.4 (−4.9 to 4.1)	.85
	Non-FGR	97.5 (16.0)	303	97.0 (15.9)	323	0.2 (−2.3 to 2.9)	

Abbreviations: FGR, fetal growth restriction; MD, mean difference; WRAT-4, Wide Range Achievement Test, fourth edition.

^a^ Scales were age standardized with a normative mean (SD) of 100 (15).

^b^ Adjusted for potential confounders (gestational age at trial entry, antepartum hemorrhage, preterm prelabor rupture of membranes, and country of birth) and clustering of children from multiple pregnancy.

**Table 4.** Parental Rating of Behavior at Midchildhood of Children Exposed to Repeated-Dose Betamethasone Treatment or Placebo

Outcome	Subgroup	Repeated-Dose Betamethasone Treatment Group		Placebo Group		Treatment Effect, MD (95% CI)	*P* Value for Interaction^a^
		Mean (SD) Score	Total No.	Mean (SD) Score	Total No.		
SDQ total difficulties score^b^	FGR	11.4 (6.7)	121	10.4 (5.5)	108	1.0 (−0.6 to 2.7)	.36
	Non-FGR	10.8 (6.9)	310	10.7 (6.7)	326	0.0 (−1.1 to 1.1)	
BRIEF global executive composite *t* score^c^	FGR	52.1 (13.0)	120	51.8 (11.5)	106	0.3 (−3.2 to 3.7)	.98
	Non-FGR	52.5 (13.0)	308	52.1 (12.8)	323	0.2 (−1.9 to 2.3)	
CADS ADHD index *t* score^c^	FGR	50.9 (6.2)	122	51.7 (6.7)	109	−0.8 (−2.5 to 0.8)	.52
	Non-FGR	51.1 (6.9)	310	51.3 (7.0)	326	−0.1 (−1.3 to 0.9)	

Abbreviations: ADHD, attention-deficit/hyperactivity disorder; BRIEF, Behavior Rating Inventory of Executive Function; CADS, Conners’ ADHD/*DSM-IV* Scales; FGR, fetal growth restriction; MD, mean difference; SDQ, Strengths and Difficulty Questionnaire.

^a^ Adjusted for potential confounders (gestational age at trial entry, antepartum hemorrhage, preterm prelabor rupture of membranes, and country of birth) and clustering of children from multiple pregnancy.

^b^ Scores range from 0 to 40, with 14 to 16 indicating borderline and at least 17, abnormal.

^c^ Mean (SD) score, 50 (10).

Regardless of treatment exposure, children with compared with those without FGR had an increased risk of death or moderate to severe disability (OR, 1.6; 95% CI, 1.1-1.4) and motor impairment (MABC total score <15th centile: OR, 1.5 [95% CI, 1.2-1.8) and had lower IQ (MD, −3.3; 95% CI, −5.8 to −0.8) and lower scores for measures of attention, executive function, and reading (eTable in Supplement 2).

## Discussion

In this secondary analysis of data from the midchildhood assessments of the ACTORDS randomized clinical trial,^11^ we found that exposure to repeated antenatal betamethasone treatment was not associated with adverse effects on survival free of any disability or on death or moderate to severe disability at 6 to 8 years of age, in children with and without FGR. Some evidence suggested a differential effect for several secondary outcomes, with better scores for selective and divided attention after exposure to repeated antenatal betamethasone in children with FGR, but poorer scores for impulse control in children without FGR. These effects were small and of uncertain clinical significance and may reflect type I error. For all other measures of neurocognitive function and learning, exposure to repeated antenatal betamethasone treatment did not alter performance in midchildhood, even in the presence of FGR.

For preterm- and term-born patients, FGR is associated with adverse neurodevelopmental outcomes in childhood and adulthood, including neurosensory disability, cognitive impairments, executive dysfunction, and emotional and behavioral difficulties.^15,32^ Imaging studies indicate that infants with FGR have abnormal structural and metabolic brain development,^33,34,35^ which may reflect suboptimal intrauterine conditions, including hypoxia-ischemia, nutritional deprivation, and/or perinatal injury. Preterm infants with FGR have decreased cortical growth^36^ and microstructural complexity, especially in the basal ganglia, brainstem, cerebellum, and frontal lobes.^37,38^ Neonatal morbidities such as chronic lung disease and necrotizing enterocolitis, which are more common in preterm infants with FGR, may exacerbate these changes.^39^

Therefore, that infants with FGR exhibit abnormal neurodevelopment is not surprising. School-aged children with FGR are reported to be more likely to have impaired social awareness, autistic mannerisms, and psychosocial issues.^40^ Concurrent with our data, cohort studies and meta-analyses have shown that children with FGR have significantly lower IQ scores and poorer overall educational achievement compared with children without FGR.^41^ By adulthood, those with FGR tend to have lower incomes because they are less likely to have professional or skilled employment.^42^ Furthermore, they also have a greater risk of adverse psychological outcomes, including schizophrenia, anxiety, and mood disorders.^43^

Although the benefits of repeated antenatal corticosteroid therapy are well established and human studies have demonstrated long-term safety,^11,44^ concerns remain about the use of this treatment in FGR, given reports from animal studies suggesting long-term adverse effects of treatment on neurosensory function.^4,45^ For example, in FGR sheep, antenatal betamethasone exposure was associated with significantly reduced expression of 5α-reductase and the subsequent concentration of the endogenous neuroprotective steroid allopregnanolone.^46^ Animal studies^4,5,7^ have also reported adverse effects, including reduced brain growth, disrupted expression of neuronal components involved with plasticity and apoptosis, and delayed glial cell maturation. Many of these studies administered corticosteroids at gestations analogous to 23 to 34 weeks’ pregnancy, a period in which severe FGR is common.^14^ On this basis, we hypothesized that repeated antenatal corticosteroid treatment may compound the adverse effects already imposed by FGR.

However, contrary to our hypothesis, we did not find any evidence of adverse effects of repeated-dose antenatal corticosteroid treatment on neurocognitive function in children with FGR. One explanation for this might be that infants with FGR appeared to have greater benefit from repeated antenatal corticosteroid therapy, with a nearly 2-fold reduction in serious neonatal morbidity. Thus, the decrease in serious postnatal complications may have counteracted any potential adverse effects of corticosteroid exposure. Cartwright et al^30^ have shown that exposure to repeated-dose antenatal corticosteroid treatment was associated with improved postnatal linear growth in children with FGR, which may have a positive influence on neurodevelopment. For example, low birth weight followed by rapid catch-up growth during infancy is associated with improved neurodevelopment at 2 years of age.^47^ Further, height in late childhood and early adulthood is positively associated with IQ,^48^ and improved linear growth during early childhood and late adolescence is independently and positively associated with later cognitive ability and educational attainment.^49^ Thus, several possible mechanisms may explain why effects on neurodevelopment in human trials may be different than those of animal studies, as Cartwright et al^30^ have shown for long-term cardiometabolic function.

One particular concern is whether use of repeated antenatal corticosteroids in FGR could increase the risk of attention-deficit/hyperactivity disorder. Attention-deficit/hyperactivity disorder is associated with altered concentration of neurotrophins, which regulate neuronal growth, morphology, migration, and apoptosis,^50,51,52,53^ and evidence suggests that neurotrophin expression is regulated by corticosteroids.^54,55^ Fetal growth restriction did not influence the parent-reported attention-deficit/hyperactivity disorder scale, but repeated betamethasone treatment had a small, positive association with the direct assessment of divided attention and possibly impulse control in children with FGR. Although a type I error cannot be excluded, this raises the possibility that treatment with antenatal corticosteroids could be neuroprotective in FGR. This possibility is supported by the finding of higher umbilical cord blood neurotrophin concentrations, such as brain-derived neurotrophic factor and neurotrophin-3, in infants exposed to antenatal corticosteroids.^56^ In infants with FGR, exposure to repeated-dose antenatal corticosteroid treatment was not associated with a change in head circumference *z* score at birth.

### Limitations

A key limitation of this study is the inherent risk of bias in subgroup analyses. Nevertheless, given the high rates of FGR among preterm infants and the ongoing concerns around efficacy and safety of repeated exposure to corticosteroid treatment in this subgroup, we believed that this exploratory analysis was important, particularly because further trials of repeated exposure to antenatal corticosteroid treatment are unlikely to be performed. Another potential source of bias in this study is the inclusion of birth weight in the definition of FGR because subgroup analysis should strictly only involve factors identified before randomization. However, we were concerned that the rate of FGR reported at trial entry (5%) underrepresented the actual degree of FGR in this high-risk cohort and could potentially obscure the effect of FGR on outcomes. We took a conservative approach, defining FGR as a birth weight less than the third centile rather than the more commonly used 10th centile, and used customized rather than population centiles because of the strong association between preterm birth and FGR. The key strengths of our study include the high follow-up rate and comprehensive assessment of participants.^11^

The findings of this study relate to single repeated doses of betamethasone and may not necessarily apply to other repeated-dose corticosteroid regimens. The relative effect of repeated-dose antenatal corticosteroid treatment at very early compared with later gestational ages, in the short and long terms, is also not known.

## Conclusions

Repeated antenatal betamethasone treatment was not associated with adverse effects on survival free of any disability or on death or moderate to severe disability at 6 to 8 years of age, even in the presence of FGR. Physicians should use repeated doses of antenatal corticosteroids when indicated before preterm birth, regardless of FGR, in view of the associated neonatal benefits and absence of later adverse effects.

## Notes

Supplement 1. **Trial Protocol**

Supplement 2. **eTable.** Primary and Secondary Neurocognitive Outcomes at Midchildhood of Children With and Without Fetal Growth Restriction (FGR)
