# Supplementary material for: Association of Fetal Growth Restriction With Neurocognitive Function After Repeated Antenatal Betamethasone Treatment vs Placebo: Secondary Analysis of the ACTORDS Randomized Clinical Trial
Source: JAMA Netw Open. 2019 Feb 1;2(2):e187636. doi: 10.1001/jamanetworkopen.2018.7636 (PMC6484607; doi:10.1001/jamanetworkopen.2018.7636)
Supplement: Supplement 1. — Trial Protocol [file jamanetwopen-2-e187636-s001.pdf]

ACTORDS
AUSTRALASIAN COLLABORATIVE TRIAL OF REPEAT DOSES OF CORTICOSTEROIDS FOR THE PREVENTION OF NEONATAL RESPIRATORY DISEASE

The Liggins Institute
An Institute of The University of Auckland

EARLY SCHOOL-AGE OUTCOMES AFTER EXPOSURE TO REPEAT ANTENATAL CORTICOSTEROIDS – A RANDOMISED CONTROLLED TRIAL

New Zealand Study Protocol

NZ Investigators

Prof. Jane Harding
Prof. Wayne Cutfield
Dr Malcolm Battin
Dr Stuart Dalziel
Dr Christopher McKinlay
Mrs Coila Bevan (Research Nurse)

Liggins Institute
University of Auckland
Private Bag 92019
Auckland Mail Centre
Auckland 1142

Phone: +64 (9) 3737599 ext 82733
Fax: +64 (9) 3737497
Email: J.Harding@auckland.ac.nz

AUSTRALASIAN COMMITTEE

Caroline Crowther
Ross Haslam
Lex Doyle
Peter Anderson
Janet Hiller
Jane Harding
Jeffrey Robinson

Maternal Perinatal Clinical Trials Unit
Department of Obstetrics & Gynaecology
University of Adelaide
Women's & Children's Hospital
72 King William Road
NORTH ADELAIDE SA 5006

Phone: +61 (8) 81617611
Fax: +61 (8) 8161 7652
Email: actords@adelaide.edu.au

CONTENTS

1 AIM 3
- 1.1 Specific Aims 3
- 1.2 Study Hypothesis 3

2 SIGNIFICANCE 3
- 2.1 Improved Outcomes for Preterm Babies 3
- 2.2 Understanding the Developmental Origins of Adult Disease 4

3 SCIENTIFIC BACKGROUND 5
- 3.1 Preterm Birth and the Role of Corticosteroids 5
- 3.2 Evidence for Single Course Antenatal Corticosteroids 5
  - 3.2.1 Time Window 6
  - 3.2.2 Childhood Outcomes 6
  - 3.2.3 Long-term Outcomes 7
  - 3.2.4 Maternal Outcomes 7
- 3.3 Trials of Repeat Course Antenatal Corticosteroids 7
  - 3.3.1 Neonatal Outcomes 8
  - 3.3.2 ACTORDS 9
  - 3.3.3 Preschool Outcomes 9
- 3.4 Current Evidence for the Beneficial and Adverse Effects of Repeat ACS 10
  - 3.4.1 Effect on Brain and Neurodevelopment 10
  - 3.4.2 Effect on Lung Function 10
  - 3.4.3 Effect on Growth & Body Composition 11
  - 3.4.4 Effect on Blood Pressure 11
  - 3.4.5 Effect on Glucose Homeostasis 11
  - 3.4.6 Effect on Cortisol 11
- 3.5 Summary 12

4 RESEARCH PLAN 12
- 4.1 Design 12
- 4.2 Subjects 12
- 4.3 Primary Outcome 12
- 4.4 Secondary Outcomes 13
- 4.5 Data management and analyses 14
- 4.6 Structure of assessments 14

APPENDIX 1: PAEDIATRIC ASSESSMENT 15

APPENDIX 2: PSYCHOLOGY ASSESSMENT 17

APPENDIX 3: PHYSIOLOGICAL STUDIES 18

REFERENCES 21

1 AIM

The aim of this study is to assess whether there are additional health gains, without adverse effects, in mid-childhood, after exposure to repeat doses of antenatal corticosteroids (ACS). We will study New Zealand children at 6-8 years corrected age who took part in the ACTORDS trial (Australasian Collaborative Randomised Trial of Repeat Doses of Corticosteroids), which randomised mothers at risk of preterm birth at less than 32 weeks, after an initial course of ACS, to either weekly corticosteroids or placebo. ACTORDS found that repeat ACS had clinically important beneficial effects on premature lung disease. However, repeat ACS were also associated with a small negative effect on fetal growth and data from animal studies suggests that increased fetal exposure to ACS may adversely affect brain development and increase the long-term risk of metabolic and cardiovascular disease. Thus the short-term benefits of repeat ACS could be offset by these other harms. There is currently no strong evidence regarding the effects of repeat ACS beyond the age of 2 years. Therefore, we will study a range of health outcomes as well as physiological variables known to be associated with the development of adult disease in order to determine if there is overall benefit from repeat ACS at 6-8 years corrected age.

1.1 Specific Aims

The study consists of three components: 1) general health and neurosensory function (paediatric assessment); 2) cognition and behaviour (psychological assessment); and 3) metabolic, endocrine and cardiovascular function (physiological studies). Parts 1 and 2 will be conducted in the entire Australasian ACTORDS cohort but part 3 will be conducted only in the New Zealand subgroup. More specifically this study will investigate the following outcomes:

table 1-1: ACTORDS NZ 6-8y Follow-up Study Outcomes

**Paediatric Assessment**	Mortality<br>Physical health<br>Blood pressure<br>Growth<br>Lung function<br>Sensorineural impairment<br>Chronic illness<br>Health related quality of life
**Psychological Assessment**	General cognitive ability<br>Attention & executive function<br>Memory & learning<br>Visual perception<br>Academic achievement<br>Behaviour
**Physiological Studies**	Ambulatory blood pressure<br>Bone mass & body composition<br>Insulin sensitivity and glucose control<br>Basal and stress cortisol<br>Renal function

1.2 Study Hypothesis

The study hypothesis is that antenatal administration of repeat ACS to women who remain at risk of preterm birth at less than 32 weeks gestation, after an initial course of ACS, has beneficial effects on their children at 6-8 years corrected age with regard to the outcomes in table 1-1.

2 SIGNIFICANCE

2.1 Improved Outcomes for Preterm Babies

This study will provide the first reliable evidence of the effects of repeat antenatal corticosteroids up to school age. This information will be of immediate clinical relevance and potentially improve outcomes after preterm birth.

Preterm birth is a major health issue in New Zealand, affecting approximately 7% of births or 4000 babies each year (New Zealand Health Information Service 2008). Despite advances in obstetric care the

proportion of babies born preterm is actually increasing, a trend that is consistent throughout the developed world (Callaghan 2006; Craig 2002; Joseph 1998; Tucker 2004). The care of these small babies consumes considerable health care resources and places significant stress on families. Prematurity is responsible for 75% of neonatal deaths (Hack 1999) and is the leading cause of infant mortality (Callaghan 2006).

ACS substantially reduce the occurrence and severity of respiratory distress syndrome (RDS), which is the principle cause of neonatal mortality and morbidity (Roberts 2006). However, there is no evidence that the benefits of a single course of ACS persist beyond 7 days. Furthermore, babies born more than 7 days after a single course of ACS may have increased mortality and morbidity (McLaughlin 2003). This is an important clinical problem as between 25% (McLaughlin 2002) and 50% (Modi 2001) of women who receive a course of ACS remain undelivered 7-14 days later but continue to have increased risk of preterm birth. These babies would be eligible for repeat ACS, which have been shown to have additional benefits for RDS and serious neonatal morbidity (Crowther 2011). However, data from both human observational and animal experimental studies suggests that increased exposure to ACS may cause both short and long-term adverse effects in various organ systems, although interpretation of these data is not straightforward.

To date, human randomised studies of repeat ACS have not extended beyond 2 years corrected age. Two of these studies found that repeat ACS were associated with small reductions in fetal growth (Crowther 2006; Wapner 2006), but the effects were rapidly reversed after birth and no differences in growth were seen at 2 years corrected age. One trial found that repeat ACS may be associated with an increased risk of cerebral palsy, although the result was not statistically significant (Wapner 2007). ACTORDS children exposed to repeat ACS had increased attention difficulties at 2 years corrected age (Crowther 2007). These findings justify caution with regard to the use of repeat ACS and long-term follow-up of randomised cohorts is urgently needed before repeat ACS can be safely recommended for routine clinical use, despite their proven short-term benefit.

ACTORDS will be the first and possibly the only randomised trial of repeat ACS to assess children at school age. The Canadian MACS trial, which recently completed recruitment, is also planning long-term follow-up but will assess children only at 5 years corrected age. However, many important outcomes, such as lung function and educational achievement and learning cannot be readily assessed until school age. This study of ACTORDS children will also be the first detailed investigation of the long-term physiologic effects of repeat ACS in humans, which is another important area of concern.

2.2 Understanding the Developmental Origins of Adult Disease

The developmental origins of health and disease (DOHaD) has become an important paradigm in the study of the pathogenesis of adult disease (Gluckman, Hanson, 2005). A central tenet of the paradigm is that organisms can undergo long-term structural or functional changes in early development in response to nutritional and other environmental cues. These long-term effects may be mediated by epigenetic changes in gene expression (nuclear or mitochondrial), altered stem cell allocation, or resetting of homeostatic mechanisms (Barker 2006; Pike 2008). Early developmental responses can contribute to later disease risk if there is a mismatch between fetal and adult environments, such that early phenotypic adaptations limit an individual’s ability to adequately respond to subsequent environmental challenges (Godfrey 2007).

Early developmental adaptions appear to occur not only in response to adversity but also within the normal range of growth and development (Barker 2006). For example, the inverse relationship between birth weight and cardiovascular mortality is continuous throughout the normal birth weight range (Seckl 2001). Increasingly, early developmental factors are being linked to other chronic illnesses including osteoporosis (Cooper 1997; Godfrey 2001), depression (Thompson 2001), schizophrenia (Gluckman, Cutfield, 2005), and obstructive respiratory disease (Lucas 2004; Shaheen 2004). There is now also evidence to suggest that early developmental adaptations can be inherited by non-genomic mechanisms (Gluckman 2007).

An important potential mechanism by which environmental factors may cause long-term effects *in utero* is fetal overexposure to maternal glucocorticoids. For example, in animals it has been shown that maternal undernutrition impairs the placental barrier to cortisol, thereby exposing the fetus to excess maternal glucocorticoids, despite normal circulating maternal cortisol levels. This in turn leads to lower

birth weight, altered postnatal hypothalamic-pituitary-adrenal (HPA) axis function and hypertension (Edwards 1993; Langley-Evans 1997; Seckl 2001). Similar changes in the offspring occur following maternal administration of synthetic corticosteroids, which are not metabolised by placental 11-beta-hydroxysteroid dehydrogenase (11βHSD) (Seckl 2001).

Given the potential association between corticosteroids and fetal programming it is important that repeat ACS receive careful longitudinal evaluation. This study will provide the first human experimental evidence of the metabolic and cardiovascular effects of repeat antenatal glucocorticoid exposure at school age. These data may offer further insights into the mechanisms underlying the developmental origins of disease.

3 SCIENTIFIC BACKGROUND

3.1 Preterm Birth and the Role of Corticosteroids

Preterm babies are at high risk of RDS due to immature lung development. Incomplete alveolar development limits the surface area available for gas exchange, while qualitative and quantitative deficiencies in surfactant cause lung collapse (Roberts 2006). RDS affects 87% of infants less than 28 weeks, 64% at 29-30 weeks, 50% at 31-32 weeks and 20% at 33-34 weeks (Boggess 2005). It is the principal cause of early neonatal mortality and morbidity and is an important risk factor for neurodevelopmental impairment. RDS is associated with cerebroventricular haemorrhage and periventricular leucomalacia, both of which contribute to cerebral palsy and other long-term disability. Severe RDS has also been linked to a global decrease in brain volume on volumetric magnetic resonance imaging (MRI) (Thompson 2007).

One of the most significant discoveries in perinatal medicine was the recognition that fetal exposure to corticosteroids induces lung maturation, especially surfactant pathways, and thus reduces the incidence and severity of RDS. Liggins and Howie pioneered this work in New Zealand in the 1960s at National Women's Hospital and conducted the first randomised controlled trial of ACS. The Auckland Steroid Trial remains the largest (1,142 mothers; 1,218 babies) and most significant study of single course ACS ever to be performed. [*] Numerous other smaller trials have confirmed the beneficial effects of ACS for RDS (Roberts 2006).

ACS are inexpensive, simple to use and produce significant benefit for babies born preterm. The NIH has concluded that ACS are 'a rare example of a technology that yields substantial cost savings in addition to improving health' (National Institutes of Health 1994). ACS have become an accepted cornerstone of modern perinatal practice. The original Liggins and Howie protocol, which administered two 12mg doses of betamethasone 24 hours apart to women at risk of preterm birth < 34 weeks gestation, continues to be the most widely used treatment regimen.[**]

3.2 Evidence for Single Course Antenatal Corticosteroids

The most recent Cochrane systematic review of single course ACS, which included 21 randomised trials and 4269 babies, found significant neonatal benefit including reductions in RDS (occurrence, severity, and respiratory support), neonatal death, cerebroventricular haemorrhage, necrotising entercolitis, and early sepsis (table 3-1) (Roberts 2006). Although 8 of these trials (848 babies) permitted repeat doses of ACS, the results of the systematic review were not affected by their inclusion. ACS showed a similar relative benefit for RDS when administered between 26 to 34 weeks gestation (table 3-1). Although no benefit was seen when administered <26 weeks gestation it is possible that this is a type 2 error given the small number of babies in this subgroup and the wide confidence intervals. Because RDS is common <32 weeks gestation the absolute benefit of ACS was large, with a number needed to benefit (NNTB) of 5 and 9 when ACS were administered between 26-29 weeks and 30-32 weeks respectively. Between 32-33

---

* A complete analysis of the Auckland Steroid Trial data was never published. The paper that is widely quoted (Pediatrics 1972;50(4):515-25) included the results of only 287 women. The remaining data was published in various conference reports. All data was extracted for the most recent Cochrane systematic review (Roberts 2006).

** After the first 717 women were recruited into the Auckland Steroid Trial the dose of betamethasone was increased to a total 48mg but no additional benefit was found.

weeks the NNTB was 15. Although ACS reduced the risk of RDS there was no effect on the risk of chronic lung disease. Current evidence is that there is no adverse effect on fetal survival or neonatal outcomes if delivery occurs within 7 days (Roberts 2006).

table 3-1: Benefits of Single Course ACS by Gestational Age at First Dose (Roberts 2006)

	All Gestations	n	<26wk	n	26-29wk	n	30-32wk	n	33-34wk	n
RDS	RR 0.66 (0.59-0.73) NNTB 8	4038	RR 2.86 (0.37-21.87)	24	RR 0.49 (0.34-0.72)	242	RR 0.56 (0.36-0.87)	361	RR 0.53 (0.31-0.91)	434
Neonatal death	RR 0.69 (0.58-0.81) NNTB 22	3956	RR 1.87 (0.61-5.72)	27	RR 0.67 (0.45-0.99)	227	RR 0.51 (0.23-1.11)	195	RR 1.11 (0.49-2.48)	339
Cerebro-ventricular haemorrhage	RR 0.54 (0.43-0.69) NNTB 21	2872	RR 1.2 (0.24-6.06)	27	RR 0.45 (0.21-0.95)	227	RR 0.23 (0.03-2)	295	RR 1.11 (0.23-5.4)	339
Necrotising enterocolitis	RR 0.46 (0.29-0.74) NNTB 30	1675								
Early Sepsis	RR 0.56 (0.38-0.85) NNTB 27	1319								

[RR= relative risk; ( ) = 95% confidence interval; NNTB = number needed to benefit; n = infants]

The Cochrane review did not show any significant effect on RDS beyond 34 weeks (35-36 weeks: RR 0.61, 95%CI 0.11-3.26, 189 babies). However, this may represent a type 2 error as other studies have shown that ACS continue to have beneficial respiratory effects at term (Stutchfield 2005). Nevertheless, ACS are not generally used >34 weeks gestation because of the low incidence of RDS.

3.2.1 Time Window

The benefits of ACS appear to be short lived. The Cochrane systematic review of single course ACS found that there was no benefit if the fetus remained undelivered 7 days after treatment with ACS (table 3-2) (Roberts 2006). Peak benefit occurred sometime after 24 hours but before 7 days. While caution is required with post hoc analysis (Gates and Brocklehurst 2007) there is currently no evidence to support benefit from ACS beyond 7 days. Furthermore, delaying delivery by more than 7 days following a course of ACS may be harmful. In the Cochrane review such fetuses were smaller at birth (mean difference in birth weight 147g) (Roberts 2006) and in another meta-analysis (862 babies) had increased neonatal mortality (RR 3.24, 95% CI 1.32-7.96) (McLaughlin 2003).

table 3-2: Benefits of Single Course ACS According to Time to Birth (Roberts 2006)

	<24hr	n	<48hr	n	24hr to 7days	n	>7days	n
RDS	RR 0.87 (0.66-1.15)	517	RR 0.63 (0.43-0.93)	374	RR 0.46 (0.35-0.6)	1110	RR 0.82 (0.53-1.28)	988
Neonatal death	RR 0.53 (0.29-0.96)	295	RR 0.49 (0.3-0.81)	339	RR 0.74 (0.51-1.07)	563	RR 1.45 (0.0.75-2.8)	561
Cerebro-ventricular haemorrhage	RR0.54 (0.21-1.36)	264	RR 0.26 (0.09-0.75)	339	RR 0.51 (0.23-1.13)	482	RR 2.01 (0.37-10.86)	453

[RR= relative risk; ( ) = 95% confidence interval; n = infants]

3.2.2 Childhood Outcomes

ACS appear to be neuro-protective, with a strong trend towards reduced developmental delay (RR 0.49, 95%CI 0.24-1) and cerebral palsy (RR0.6, 95%CI 0.34-1.03) (Roberts 2006). An observational study also found that single course ACS were associated with a higher intelligence quotient (WISC-3 full scale mean difference 6.2, 95%CI 0.8-11.6) at age 14 years in very low birth weight children (Doyle 2000). However, follow-up of a randomised cohort at age 6 years (304 children), found that ACS exposed children had lower Raven Progressive Matrices scores (mean difference 1.2 or 0.3SD, p=0.05), which is a test of general intelligence or abstract thinking (MacArthur 1982). The effect was greatest in boys (mean difference 1.6, p=0.01). This finding could not be replicated in a Dutch follow-up study at 10-12 years (Schmand 1990), although the study was small (90 children; follow-up rate 88%) and not powered to detect clinically important differences in intelligent quotient (IQ).

In an observational study ACS increased systolic and diastolic blood pressure at age 14 years (Doyle 2000) but a randomised study showed no effect at age 6 years (Dalziel 2004). Childhood growth is unaffected by single course ACS (Roberts 2006). Lung function has been assessed only in an observational study but was also unaffected (Doyle 2000).

3.2.3 Long-term Outcomes

The use of single course ACS for preterm birth <34 weeks has not been shown to cause any long-term clinical harm, at least into early adulthood. Follow-up of a Dutch randomised cohort at age 20 years (81 neonatal survivors; follow-up rate 74%) found that ACS had no effect on growth, blood pressure, cognition, psychopathology, puberty onset, sexual function, education, or socioeconomic status (Dessens 2000). Thirty year follow-up from the Auckland Steroid Trial (534 neonatal survivors; follow-up rate 56%) similarly showed no differences in blood pressure, obesity, cholesterol, morning cortisol, lung function, body size, bone mass, employment status, IQ, concentration, psychiatric morbidity or health related quality of life (Dalziel, Lim, 2005; Dalziel, Walker, 2005; Dalziel, Fenwick, 2006; Dalziel, Rea, 2006; Dalziel 2007). However, ACS exposed subjects showed evidence of insulin resistance on oral glucose challenge, although there was no difference in the incidence of diabetes (Dalziel, Walker, 2005). The clinical significance of this finding for later adulthood remains unknown, but insulin resistance is a risk factor for the later development of diabetes and cardiovascular disease.

3.2.4 Maternal Outcomes

The Cochrane review found that maternal outcomes are unaffected by single course ACS (Roberts 2006), although the analysis by McLaughlin (2003) found increased rates of chorioamnionitis if delivery did not occur before 7 days (RR2.91, 95%CI 1.25-6.74). Single course ACS are safe and beneficial in women with premature rupture of membranes (Harding 2001; Roberts 2006) and pregnancy related hypertension syndrome (Roberts 2006).

3.3 Trials of Repeat Course Antenatal Corticosteroids

Liggins and Howie recognised in the first report of the Auckland Steroid Trial (Liggins 1972) that ACS did not appear to have benefit for RDS beyond 7 days and suggested that there may be benefit in giving repeat doses. In some centres this became standard practice and by the late 1990s the use of repeat doses was quite widespread (Brocklehurst 1999; Quinlivan, Evans, 1998) despite a lack of strong evidence to support their use. Although some animal studies suggested that repeat doses may be more effective, for example, improving lung function in sheep (Ikegami 1997), reports of efficacy in humans were conflicting (Banks 1999; Elimian 2000). A body of evidence also started to emerge that raised concerns about the safety of exposing fetuses to higher doses of glucocorticoids. Animal models demonstrated fetal growth restriction (Ikegami 1997; Quinlivan, Archer, 1998), impaired cerebral development (Dunlop 1997; Huang 1999), altered hypothalamic-pituitary-adrenal axis (Ikegami 1997) and emphasematous alveolar development (Tschanz 1995; Willet 2001). Observational data in humans also pointed to impaired fetal growth (Abbasi 2000; Banks 1999; French 1999), increased perinatal mortality (Banks 1999), altered brain maturation (Modi 2001), poorer neurodevelopmental outcome (Spinillo 2004), and hyperactivity (French 2004).

In 2000 the NIH called for a moratorium on the use of repeat ACS until there was adequate evidence from randomised trials (National Institutes of Health 2000). Four randomised trials of weekly ACS have now been completed: one in Australasia (ACTORDS) (Crowther 2006), two in the United States of America (USA) (Guinn 2001; Wapner 2006), and one in Canada (Murphy 2007 abstract). ACTORDS and the National Institute of Child Health and Human Development (NICHHD) Maternal Fetal Medicine Network (MFMN) trial have both completed 2-year follow-up studies (Crowther 2007; Wapner 2007). Guinn et al have not conducted any long-term follow-up. Both American trials were terminated early because of concerns about neurological harm (Guinn 2001) and impaired fetal growth (Wapner 2006) combined with small absolute benefits and difficulties with recruitment (Wapner 2006).

table 3-3: Randomised Controlled Trials of Repeat ACS

	Guinn 2001	Wapner / MFMN 2006	Crowther / ACTORDS 2006	Murphy/ MACS 2007
Country	USA	USA	Australia & NZ	Canada
Study dose	Betamethasone 24mg weekly <34wk	Betamethasone 24mg weekly <34wk	Betamethasone 11.4mg weekly <32wk	Fortnightly <34 wk
Calculated sample size	1000	2400	980	1900
Total women recruited	502	495	982	1858
Mean gestation at entry	28	28	28	
Mean gestation at delivery	33	34	32	
> 3 study doses	40%	62%	25%	
Paediatric follow-up	? none	2yr (completed)	2yr (completed)<br>6-8yr (this protocol)	2yr, 5yr

Two small pilot studies have also been published; one in Canada (Aghajafari, Murphy, Ohlsson, 2002, 12 women) and the other in the USA (McEvoy 2002, 37 women). Another trial was started in the United Kingdom, TEAMS (trial of the effects of antenatal multiple courses of steroids versus a single course), but was stopped in 2003 after recruiting 154 women, because of inadequate funding. There are also two randomised trials examining the effect of giving only a second course of ACS. One of these studies, which used a rescue protocol, was terminated early because of increased severity of RDS in the treatment arm (Peltoniemi 2007). Another trial by Obstetrix Medical Group, USA, which gives the second course of ACS after 14 days, is ongoing (NCT00201643).

3.3.1 Neonatal Outcomes

The current Cochrane systematic review of repeat ACS (2028 women) includes ACTORDS, the two USA trials and the pilot studies by Aghajafari and McEvoy (Crowther 2011). This review found that the administration of weekly ACS to women at risk of preterm birth <34 weeks gestation reduced all RDS, severe RDS and a composite outcome of serious morbidity, including chronic lung disease, severe cerebroventricular haemorrhage, necrotising enterocolitis, and periventricular leucomalacia (table 3-4). However, there was no effect on individual morbidity outcomes. In keeping with the decrease in RDS, repeat ACS also reduced oxygen and surfactant use, and patent ductus arteriosus (PDA) requiring treatment but not other respiratory support parameters. There were no differences in fetal and neonatal mortality, neonatal or maternal infection, or chronic lung disease.

table 3-4: Benefits of Repeat ACS (Crowther 2011)

	Treatment Effect	n
RDS	RR 0.82 (0.72-0.93)<br>NNTB 17	2155
Severe RDS	RR 0.6 (0.48-0.75)<br>NNTB 15	2139
Serious morbidity	RR 0.79 (0.67-0.93)<br>NNTB 21	2157

[RR= relative risk; ( ) = 95% confidence interval; NNTB = number need to benefit; n = infants]

There were no differences in mean birth weight, head circumference, or length. However, two trials showed small reductions in fetal growth in the repeat ACS exposed groups: ACTORDS found lower mean Z-scores for birth weight and head circumference (absolute difference 0.13 and 0.17 respectively); the MFMN trial found an increase in small for gestational age (<10th percentile) babies, which in post hoc analysis was confined to those infants receiving 4 or more study doses. Both trials reported rapid neonatal catch-up growth so that no differences in size were seen at time of discharge.

3.3.2 ACTORDS

The Australasian Collaborative Trial of Repeat Doses of Corticosteroids (ACTORDS) assessed whether repeat doses of ACS, given to women who remained at risk of preterm birth at less than 32 weeks gestation, reduced the risk of neonatal respiratory disease without adverse effects (Crowther 2006). Women were recruited to the trial if they had ongoing risk of preterm birth 7 or more days after an initial course of ACS and were <32 weeks gestation. Women were excluded if they were in the second stage of labour, had chorioamnionitis needing urgent delivery, had mature lung development, or if further corticosteroid therapy was judged to be essential. Approximately 44% of the eligible women who were asked to take part in the study gave consent. Participants were randomised to weekly doses of betamethasone 11.4mg (celestone chronodose) (n = 489) or saline placebo (n = 493), continuing up to 32 weeks gestation. Randomisation was stratified by centre, gestation, and number of fetuses. Of 1146 infants alive at randomisation there were 1090 survivors to initial discharge home, of whom 342 were from New Zealand. The mean gestational age of both groups at initial steroids was 26 weeks, and at trial entry and birth it was 28 and 32 weeks respectively.

ACTORDS found that repeat doses of ACS had a beneficial effect on respiratory morbidity and PDA (table 3-5). There was no effect on survival, chronic lung disease or other morbidity but the repeat ACS group had reduced combined serious morbidity, defined as air-leak syndrome, PDA, oxygen use at 36 weeks postmenstrual age, severe cerebroventricular haemorrhage, periventricular leukomalacia, necrotizing enterocolitis, or retinopathy. Repeat ACS had a small negative effect on fetal growth (section 3.3.1). No other fetal or neonatal adverse clinical outcomes occurred.

The hypothalamic-pituitary-adrenal (HPA) axis was studied in two subgroups of infants. In Adelaide cord serum cortisol concentration was measured in 67 infants and salivary cortisol was studied in 51 infants (Ashwood 2006). No significant difference in mean cord serum cortisol concentration was found between those exposed to repeat ACS (34 infants) and those exposed to a single course of ACS (33 infants) (mean difference -26 nmol/L [95%CI 57, 5 nmol/L], p=0.10). Basal morning salivary cortisol concentration was lower in the repeat ACS group on day 7 of life (median 11.7 [18 infants] v. 18.2 [21 infants] nmol/L, p=0.04), but not on day 3, 14 or 21. Stress salivary cortisol was lower in the repeat ACS group on day 3 (median 11.9 [11 infants] v. 21.4 [16 infants] nmol/L, p = 0.02). In an Auckland subgroup no difference in morning plasma cortisol concentration was seen between those exposed to repeat ACS (30 infants) and those exposed to single course ACS (33 infants) on day 2 of life (249 v. 265 μmol/L) (Battin 2007). Some of these babies also received a metyrapone challenge, which tests the whole HPA axis. However, no differences in plasma cortisol or adrenocorticotropin (ACTH) concentrations were seen between the groups 3 hours after administration of metyrapone (17 v. 9 infants).

table 3-5: Benefits of Repeat ACS in ACTORDS (Crowther 2006)

	Repeat ACS n 567	Placebo n 577	Adjusted RR (95% CI)	NNTB (95% CI)
RDS	186	239	0.82 (0.71-0.95)	13 (8-48)
Severe RDS	65	114	0.6 (0.46-0.79)	13 (9-24)
Surfactant use	24	32	0.81 (0.68-0.97)	95 (56-601)
PDA	40	67	0.59 (0.4-0.87)	21 (14-66)
Serious morbidity	20	26	0.79 (0.65-0.97)	106 (63-740)

Mothers in the repeat ACS group had a higher rate of caesarean section (RR 1.13, CI95% 1.02-1.24) but the overall rate of caesarean section in the trial was high at 62%. There were no differences in other maternal outcomes.

3.3.3 Preschool Outcomes

To date only two trials have reported outcomes beyond the neonatal period. ACTORDS found that infants exposed to repeat ACS were more likely to be rated by parents as intense (3.9 [SD 0.6] v. 3.8 [SD 0.7], p=0.03) and negative in mood (3.2 [SD 0.6] v. 3.1 [SD 0.7], p=0.03) on the Toddler Temperament Scale (Sewell 1988) at 12 months of age (Data presented to the Perinatal Society of Australia and New Zealand, 2006). Follow-up at 2 years corrected age, which included 97% of neonatal survivors, showed that repeat ACS had no effect on survival free of neurosensory disability, developmental quotient (Bayley Scales of Infant Development), body size, blood pressure, respiratory morbidity, use of health services, or child

behaviour scores. However, there was an increase in attention problems in those exposed to repeat ACS (6% v. 3.2%, adjusted RR 1.87 [95%CI 1.03-3.42]) (Crowther 2007).

The MFMN trial performed a similar follow-up at 2 years corrected age, assessing 83% of neonatal survivors, and also found no differences in growth, developmental quotient (Bayley Scales of Infant Development), and blood pressure. There was, however, a trend to reduced risk of asthma in the repeat ACS group (8.3% v. 14.4%, RR 0.6 [0.3-1.0], p=0.05).

The MFMN 2 year follow-up study also found a trend to increased risk of cerebral palsy in the repeat ACS group, although the result was not statistically significant (2.9% v 0.5%, RR 5.7 [CI95% 0.7-46.7], p=0.12) and most of the cases of cerebral palsy occurred in those children that received 4 or more study doses (Wapner 2007). This may be a type 1 error given the low absolute event count (6 v. 1 cases of cerebral palsy) and the use of pregnancy as the denominator rather than babies, which is known to overestimate the incidence of outcomes (Gates 2007).

3.4 Current Evidence for the Beneficial and Adverse Effects of Repeat ACS

Corticosteroids can have potent effects in multiple organ systems due to their action as transcription regulators and also through the inhibition of cell growth and DNA replication. Normally the fetus is protected from exposure to maternal cortisol by placental inactivation via 11βHSD. However, synthetic steroids, such as betamethasone, are a poor substrate for 11βHSD and readily cross the placenta. Fetal tissues are particularly sensitive to corticosteroids, whether natural or synthetic, and corticosteroid exposure in early development can potentially cause long-term physiological changes. Therefore, it is essential that studies of repeat ACS include long-term follow-up of health outcomes.

The clinical evaluation of ACS is complicated by the fact that prematurity itself is associated with long-term physiological effects including elevated blood pressure (Doyle 2003; Irving 2000; Leon 2000), altered body composition (Fewtrell 2004; Uthaya 2005), impaired lung function (Anand 2003; Doyle 2006; Galdes-Sebaldt 1989; Stick 2000) and insulin resistance (Hofman 2004). Therefore, the effects of ACS can be reliably evaluated only in randomised trials, which seek to overcome the effect of confounding variables such as gestation and size at birth at birth.

While current evidence shows that single course ACS do not cause any clinical adverse outcome into early adulthood, the same cannot be assumed for repeat course ACS. There is considerable evidence from animal studies to support a dose-response relationship, although this cannot be assumed to apply in human pregnancy (Aghajafari, Murphy, Matthews, 2002).

3.4.1 Effect on Brain and Neurodevelopment

The reductions in premature lung disease associated with the use of repeat ACS could potentially lead to improved neurodevelopmental outcomes, although this benefit may be offset by possible adverse effects of repeat ACS on fetal brain development. In sheep, ACS have multiple effects on brain growth, including reduced brain size (Huang 1999), delayed myelination (Dunlop 1997; Huang 2001) and decreased long-term brain mass (Moss 2005). These effects are greatest following repeat ACS (Huang 1999; Moss 2005). Guinea pigs and rats exposed to high doses of ACS have altered behaviour into adulthood, including increased anxiety, and impaired learning and memory (Owen 2005; Welberg 2001).

In human observational studies, repeat ACS are associated with reduced head circumference (French 1999). Studies of long-term neurological outcome are conflicting with some studies showing increased risk of cerebral palsy (Shinwell 2000; Takahashi 2005) and developmental delay (Esplin 2000), but others show no effect (Hasbargen 2001; Kumar 2004; Thorp 2001) or even a reduction in the incidence of cerebral palsy (French 2004). However, the latter study also reported increased aggressive, destructive and hyperkinetic behaviour in childhood. Human randomised studies have currently only assessed the effects of repeat ACS up to 2-3 years corrected age (section 3.3.3). While these studies found that repeat ACS had no effect on developmental quotients, developmental testing at this age has limited predictive value for later cognitive ability (Rose 2003). Therefore, assessment at school age is needed to assess the overall effect of repeat ACS on neurodevelopment.

3.4.2 Effect on Lung Function

Experimental evidence in both animals and humans shows that improvements in respiratory compliance and pulmonary function can be repetitively induced despite prior treatment with corticosteroids (Ikegami 1997; McEvoy 2000; Stewart 1998). This suggests that repeat ACS may cause structural maturation of

the fetal lung in addition to stimulation of surfactant pathways. However, animal models have shown that this can also lead to emphysematous-like alveolae (Tschanz 1995; Willet 2001). The effect of repeat ACS on later lung function in humans has not been assessed in randomised studies.

3.4.3 Effect on Growth & Body Composition

A dose-dependent reduction in growth has been well documented in fetal lambs exposed to increasing doses of antenatal betamethasone (Fowden 1996; Ikegami 1997). Permanent changes in body composition have been seen in rats exposed to prenatal dexamethasone, with increased susceptibility to later obesity (Cleasby 2003). Bone cortical thickness is also markedly reduced in adult rats after antenatal exposure to dexamethasone (Swolin-Eide 2002).

In humans, repeat ACS have been associated with small reductions in fetal growth but rapid catch-up growth is achieved post-natally (section 3.3.1). At 2-3 years of age no differences in size have been detected in both observational (French 1999) and randomised (Crowther 2007; Wapner 2007) studies. There are no randomised data on the effects of repeat ACS on growth and body composition at school age.

3.4.4 Effect on Blood Pressure

ACS can cause long-term cardiovascular changes. Sheep exposed to a single course of ACS in early gestation have life-long hypertension (Dodic 1998), as do rats exposed to dexamethasone in late gestation (Levitt 1996).

In humans, single course ACS have been associated with late elevation of blood pressure in observational (Doyle 2000) but not randomised studies (Dalziel 2004; Dalziel, Walker, 2005). One observational study showed an association between repeat ACS and elevated neonatal blood pressure (Mildenhall 2006). However, in randomised studies repeat ACS have not been shown to affect blood pressure at 2 years corrected age (Crowther 2007; Wapner 2007). Nevertheless, long-term follow is needed to exclude possible late cardiovascular effects of repeat ACS.

3.4.5 Effect on Glucose Homeostasis

Insulin resistance describes a state in which target tissues have decreased responsiveness to the actions of insulin on glucose metabolism. It is associated with an increased risk of subsequent glucose intolerance and diabetes (Barker 2005). In rats ACS lead to long-term impairment of glucose tolerance in the offspring (Seckl 2001). Similarly, sheep exposed to repeat doses of betamethasone have altered insulin secretion and increased hepatic glucose-6-phosphatase activity as adults; changes that are consistent with impaired glucose tolerance in later life (Sloboda 2005).

In humans, prematurity itself causes insulin resistance and this effect is compounded by other factors such as rapid early weight gain (Regan 2006). A single course of ACS has also been shown to increase this risk in early adulthood (section 3.2.3). It is possible that repeat ACS may have an even greater effect on the insulin-glucose axis.

3.4.6 Effect on Cortisol

Administration of corticosteroids results in transient down-regulation of the hypothalamic-pituitary-adrenal (HPA) axis, due to negative feedback at the level of the hypothalamus and pituitary. The duration of this suppression after postnatal dexamethasone in preterm babies is often several weeks, and may be sufficiently severe to warrant cortisol supplementation in times of stress (Ford 1997; Ng 1997). In experimental animals, the effects of ACS exposure are complex. Rats exposed *in utero* to dexamethasone have evidence of long-term up-regulation of the HPA axis into adulthood, at least in part due to permanent down-regulation of the hippocampal glucocorticoid receptors leading to resetting of the "set point" of the negative feedback loop. Sheep exposed *in utero* to repeat doses of betamethasone have up-regulation of the HPA axis at one year of age (early adulthood) (Sloboda 2002) but this wanes over time, so that by 3 years (middle age) there is evidence of HPA axis suppression (Sloboda 2007).

The effect of ACS on HPA axis function in humans has not been well studied. In two small observational studies of preterm infants, antenatal betamethasone reduced the cortisol response to a physiological stressor at 1 week (Davis 2004; Davis 2006) and at four to six weeks of age (Davis 2006). Long-term follow-up of subjects from the Auckland Steroid Trial did not show differences in fasting plasma cortisol levels at age 30 years (Dalziel, Walker, 2005).

Data on the effect of repeat ACS on the HPA axis in humans are sparse. Studies in subgroups of babies from ACTORDS found that repeat ACS were associated with a small temporary reduction in basal salivary cortisol concentration in the first week after birth and decreased stress responses on day 3 of life (section 3.3.2). However another study found no differences in plasma cortisol and ACTH or response to metyrapone challenge at 2-3 days of age (Battin 2007). No other randomised data are available on the effects of repeat ACS on HPA axis function in humans.

3.5 Summary

This study of early school-age outcomes in ACTORDS children is justified for the following reasons:

- Repeat doses of antenatal corticosteroids (ACS) provide clinical important respiratory and other benefits for babies born preterm.
- There are no randomised data regarding the effects of repeat ACS beyond 2 years corrected age, but other evidence from animal and human non-randomised studies show that repeat ACS may be harmful in the long-term with regard to neurodevelopment, growth, and cardiovascular and metabolic disease.
- These possible effects of repeat ACS cannot be fully evaluated before school age and long-term follow is essential to assess overall safety.
- ACTORDS is currently the largest published randomised trial of repeat ACS and the only trial planning school-age follow-up.
- The results of this study will directly influence clinical practice and potentially improve outcomes for preterm babies.
- The additional physiological studies to be performed in the New Zealand subgroup will provide important direct human experimental data regarding the glucocorticoid hypothesis for the developmental origins of adult disease.

4 RESEARCH PLAN

4.1 Design

This is a follow-up study from a randomised placebo controlled trial. Children will be assessed without reference to any previous results and study personnel will be blinded to treatment group. All ages will be corrected for gestation at birth as even at 8 years correction for prematurity results in elimination of a small but potentially important bias in cognitive test scores (Rickards 1989).

4.2 Subjects

All surviving children from the ACTORDS trial alive at 6-8 years corrected who reside in New Zealand will be eligible and will be invited to participate in this study. There will be no exclusion criteria.

Women were eligible for the ACTORDS trial if they had a singleton, twin or triplet pregnancy at less than 32 weeks gestation, 7 or more days after an initial course of ACS, and had no contraindications to the use of further corticosteroids (section 3.3.2).

The initial consent for the ACTORDS trial included follow-up to 2-years corrected age. New consent will be sought for this study. It has received ethical approval from the Multi-Regional Ethics Committee of New Zealand (MEC/07/07/101).

In the New Zealand subgroup 342 infants survived to discharge from hospital and at 2 years corrected age 337 were known to still reside in New Zealand. The ACTORDS study group, based in Adelaide, has had ongoing contact with these families since the 2-year follow-up and has maintained a central database. Currently there are 307 New Zealand children on the database with 153 in Auckland, 94 elsewhere in the North Island and 60 in the South Island. The children are currently aged between 3 to 8 years. Families will be contacted when the child is between 6 to 8 years corrected age and invited to join the study. Assessments will begin in early 2008 and continue until the end of 2010 when the youngest children turn 6 years.

4.3 Primary Outcome

The primary outcome will be survival free of sensorineural disability. This will include: cerebral palsy, hearing impairment requiring a hearing aid, blindness or IQ less than 1 SD below the mean.

Cerebral palsy (CP) will be defined as a non-progressive loss of motor function with disordered tone or tendon reflexes. The presence of mild upper motor neuron signs in the absence of functional limitation will not be diagnosed as CP. CP will be classified by type and limb distribution (Cans 2007) and graded into three levels of severity based on gross motor function:

- Mild CP: ambulant with little limitation
- Moderate CP: ambulant with substantial limitation
- Severe CP: non-ambulant.

Gross motor function will also be classification according to the system of Palisano (1997).

Blindness will be defined as visual acuity less than 6/60 in the best eye after best possible correction.

The level of neurosensory disability will be further classified as follows:

- Severe disability: any of severe cerebral palsy, IQ less than -3 SD below the mean, or blindness
- Moderate disability: any of moderate cerebral palsy, deafness, or IQ from -3 SD to -2 SD below the mean
- Mild disability: mild cerebral palsy or IQ from -2 SD to -1 SD below the mean

4.4 Secondary Outcomes

Paediatric

- Mortality
- Growth: height, weight, head circumference, mid arm circumference (non-dominant), upper: lower segment ratio
- Sensorineural impairment including blindness, deafness and cerebral palsy as defined above
- Movement ABC-2 score
- Blood pressure as measured by an oscillometric device: systolic, diastolic and mean arterial pressure and proportion in the hypertensive range ($\geq 95^{th}$ percentile or 1.65 SD)
- Lung function as measured by flow spirometry: FVC, FEV1, FEV1/FVC ratio, PEF, FEF25-75, FEF50, FEF25 as % predicted for age, gender, and height
- Health related quality of life: parents will complete the Child Health Questionnaire (CHQ) and the Multi-attribute Health Status (MAHS) questionnaire
- Chronic illness, health services utilisation, and reasons for use

Psychometric

- General cognitive ability as assessed by the Weschler Abbreviated Scale of Intelligence (WASI)
- Attention & executive function as assessed by subtests from the Test of Everyday Attention for Children (TEACh), the Rey Complex Figure (RCF), the Fruit Stroop task. Parents and teachers will complete the Behavior Rating Inventory of Executive Function (BRIEF).
- Memory & learning as assessed by the Rey Auditory Verbal Learning Test (RAVLT)
- Visual perceptual skills as assessed by subtests of the Test of Visual-Perceptual Skills 3rd edition (TVPS-3)
- Academic achievement as assessed by the Wide Range of Achievement Test (WRAT4)
- Behaviour problems: parents and teachers will complete the Strength and Difficulties Questionnaire (SDQ), and the Conner's ADHD/DSMIV Scales (CADS)

Physiological

- Oscillatory ambulatory blood pressure (ABP) monitoring, 24 hour recording:
    - 24-hour, daytime, and nighttime mean ABPM parameters (systolic and diastolic BP, mean arterial BP, heart rate) and Z-scores (gender and height specific)
    - Blood pressure load and nocturnal dipping
- Frequently sampled IV glucose tolerance test (FSIGTT) with minimal model analysis (MINMOD):
    - Insulin sensitivity index (S_I)
    - Acute insulin response
    - Glucose effectiveness (Sg)
    - Glucose disappearance coefficient (Kg)
- Whole body dual energy x-ray absorptiometry (DEXA):
    - Bone area (BA), bone mineral content (BMC), areal bone mineral density (aBMD)
    - BA for height and BMC for BA and height Z-scores (gender specific)

    - Fat mass (FM) and lean mass (LM), FM percentage, FM index (FM/height)
- Hypothalmic-Pituitary-Adrenal (HPA) axis function:
    - Basal diurnal salivary cortisol and dehydroepiandrosterone (DHEA)
    - Fasting & stress plasma cortisol with FSIGTT
- Renal function
    - Creatinine
    - Estimated creatinine clearance (CrCL, ml/min/1.73m^2)

4.5 Data management and analyses

Statistical analysis

Data will be entered into a database and analysed using SAS statistics software with the assistance of the ACTORDS study statistician. Dichotomous outcome data will be contrasted by $\chi 2$ analysis or log-binomial regressions to adjust for confounding variables, and continuous data by t-test or analysis of variance (ANOVA) to adjust for confounding variables. Confounding variables will comprise sociodemographic variables, such as ethnicity, language spoken at home, family structure, mother's marital status, social class, and mother's and father's education, as well as gender. Adjustment will also be necessary to allow for a small design effect caused by non-independence of children from multiple pregnancies. P-values <0.05 will be considered statistically significant.

Power and Clinical Significance

The best estimate of the primary outcome is 87% survival free of neurosensory disability. This is based on data from the 2-year ACTORDS follow-up. Assuming 90% follow-up and a 2-sided significance level of 5%, the New Zealand subgroup (values for the total Australasian ACTORDS study cohort in brackets) will have 80% power to detect differences in the primary outcome from 87% up to 96% or down to 75% (92% to 80%); differences in cerebral palsy from 4% up to 12% or down to 0% (8% to 1%); and for outcome variables that are continuous and normally distributed (measures of growth, blood pressure, psychological tests), differences as small as 0.34SD (0.18SD). In the case of DEXA, if 200 children are scanned, the study will have 80% power to detect differences of 0.4SD, assuming DEXA parameters are normally distributed. The power of the study to detect larger differences would, of course, be higher.

For lung function the total Australasian cohort will be able to detect differences between groups of 3 to 4% for values of FVC and FEV_1 (Dalziel, Rea, 2006).

For insulin sensitivity, based on data in normal children of this age, the study will have 80% power to detect a 10% difference if 50% of children agree to this test and a 20% difference if only 25% agree.

These minimum absolute differences are considered clinically important to detect. For IQ, 0.3 SD represents a 5-point difference in IQ. A 0.3 SD decrease in head circumference in childhood is associated with a 0.7 reduction of IQ points (Gale 2004). For BMI, a 0.3 SD increase in childhood is associated with an 8% increase in the hazard ratio for death from coronary heart disease (Eriksson 1999). For insulin resistance, the risk of developing type 2 diabetes is reported to be 46% over 25 years in a high-risk population (Martin 1992).

4.6 Structure of assessments

Wherever possible, in order to maximise participation rates, all assessments will occur on the same day. The exceptions will occur when DEXA is not available on the assessment site, necessitating a separate appointment, ambulatory blood pressure measurement, which will occur overnight after the other assessments, and salivary cortisol, which will be collected over 3 typical days.

Children agreeing to the glucose tolerance test will be asked to fast overnight, and this test will be done first in the early morning. The remaining physical assessments and DEXA will then be completed after the child has had a break and breakfast. A longer lunchtime break will then be followed by the psychological assessments. Children not consenting to blood tests will not be fasted and will start the assessment later in the morning, but follow the same sequence. The entire glucose tolerance test, including setting up, takes approximately 2h, during which the child watches a movie of their choice. DEXA scanning takes <5 minutes. Physical assessment with the paediatrician, including blood pressure and lung function, takes approximately 1 hour. The psychological assessment will take 2.5 to 3 hours, and at least one break will be provided.

APPENDIX 1: PAEDIATRIC ASSESSMENT

Growth

Height and sitting height will be measured by a stadiometer to the nearest 0.1cm (bare feet), weight by electronic scales to the nearest 0.1kg (minimal clothing) and head circumference and mid-arm circumference (non dominant arm at the half-way point between the acromion to olecranon) by tape measure to the nearest 0.1cm. Values will be computed, using corrected age, for the relevant percentile, percent of median, and standard deviation scores from the British Growth Reference (Cole 1995; Cole 1998; Freeman 1995).

Blood pressure

Blood pressure (BP) will be recorded in the right arm with the subject seated in a chair after a 10-minute rest using a Dinamap automated oscillometric device. Three measurements will be made and the average systolic, diastolic and mean arterial pressure will be converted to Z-scores (age, height and gender specific) (Rosner 1993). If the child is hypertensive (>95th percentile, or approximately >110/75mmHg at 6-8 years) a second set of measurements will be taken later in the assessment. American Heart Association guidelines for the correct measurement of blood pressure and selection of cuff size will be followed (Pickering 2005). As a rule of thumb the cuff width should be approximately 75% of arm length (acromion to olecranon distance).

Lung function

Ventilatory capacity will be measured by spirometry using the EasyOne 2001 flow spirometer (NDD Technologies), which conforms to both American Thoracic Society (ATS) and European Thoracic Society (ERS) diagnostic spirometry standards. The following parameters will be assessed using forced expiration: forced vital capacity (FVC), forced expiratory volume in 1 second (FEV_1), peak expiratory flow (PEF), flow rate at 50% and 25% vital capacity (FEF_{50}, FEF_{25}), mean flow between 25-75% of vital capacity ($FEF_{25-75\%}$). ATS 1994 spirometry guidelines will be observed (American Thoracic Society 1995), which defines a satisfactory test as 3 technically acceptable trials including 2 reproducible trials (<0.2L variation in the 2 largest FVC and FEV1 measures). Therefore, a minimum of three forced expiratory manoeuvres will be performed.

The largest FVC and FEV1 from all acceptable trials will be recorded, even if they do not come from the same trial, as recommended by the ATS. Other measures will be obtained from the best trial (largest sum of FVC and FEV1) that meets acceptability criteria. Results will be expressed as percent predicted for gender, age and height for healthy, normal birth weight, Caucasian Australian children (Hibbert 1989). The use of an exponential regression form (see appendix 6.4) has ensured that percent predicted is independent of height for each of the lung function parameters. Raw data will not be corrected for ethnicity but later adjustment may be required if ethnic composition differs markedly between study groups.

The EasyOne spirometer has been shown to have very stable calibration in clinical use and to not require regular volume calibration (Pe´rez-Padilla 2006; Walters 2006). However, a study investigator will serve as a biological control to monitor spirometer performance on a regular basis. FVC or FEV1 values outside a 95% confidence interval will indicate the need for volumetric calibration (Johns 2003 p21-22).

Neurosensory function

Visual acuity will be measured using a 3m Snellen chart testing both binocular and monocular vision. Corrective lenses will be worn and the child's eye will be occluded by patch or by the examiner. Visual defects will be deemed 'severe disability' if felt to be subjectively so by the paediatrician but 'legally blind' will be defined as visual acuity <6/60 in the best eye. Further assessment of vision will be advised if fewer than 4 of 6 correct letters are read on the 6/9 line or if there is a 2-line difference between the eyes, as recommended by the American Academy of Pediatrics (2003).

Otoscopy will be performed and hearing will be screened using low and high tone whispered numbers. Children will be referred for audiology if they are considered to have language delay or if deafness is suspected. Deafness is defined as hearing impairment requiring hearing aids.

Cerebral palsy, defined as a non-progressive loss of motor function with disordered tone or tendon reflexes, will be determined by neurological examination and classified and graded as outlined above.

Motor function will also be assessed using the second edition of the Movement Assessment Battery for Children (Movement ABC-2) (Henderson 2007). Children born preterm are at increased risk of motor difficulties, even in the absence of signs of neurological impairment. The Movement ABC is a sensitive test of motor dysfunction in this group (Jongmans 1998). Age adjusted percentiles and Z scores will be determined for the three test components (manual dexterity, aiming and catching, and balance) and the total test using the reference tables in the ABC-2 manual. The ABC-2 has been standardized in a large sample of representative British children. Scores below the 15th percentile indicate motor impairment and those below the 5th percentile are associated with severe motor coordination problems.

Pubertal Status

The onset of puberty will be determined by breast development in girls and testicular volume via an orchidometer in boys (>4ml or more). Pubertal progression will be classified by Tanner stage (Marshall 1969; Marshall 1970).

General health

A paediatrician will formally assess all children by general history and physical examination to determine the presence of any significant chronic illness. Data regarding hospital readmissions will be confirmed, where necessary. The child's caregiver will be asked to complete a questionnaire relating to any respiratory morbidity, history of illness or injury and health service utilization.

Health-related quality of life

Health-related quality of life will be measured using a paediatric adaptation of a multi-attribute health status (MAHS) classification system (Saigal 1994). The MAHS classification system derives from the oncology literature and describes both the type and severity of functional limitations according to seven attributes: sensation, mobility, emotion, cognition, self-care, pain, and fertility. Each attribute has four or five levels of function. The MAHS clearly assesses outcomes other than neurological function.

Children will also be assessed with the Australian Authorised Adaptation of the Child Health Questionnaire (CHQ) (Waters 2000). The CHQ has recently been standardised on over 5000 Australian children aged 5-18 years and provides an assessment of a child's psychosocial health, physical health and well-being.

APPENDIX 2: PSYCHOLOGY ASSESSMENT

General cognitive ability

Children will be assessed using the Wechsler (1999) Abbreviated Scale of Intelligence (WASI). An estimated IQ score reflecting general intellectual ability, will be derived from four subtests: Vocabulary, Similarities, Block Design, and Matrix Reasoning. Each scale/index is age standardised. Intellectual impairment will be classified as follows:

- Mild intellectual impairment will be an IQ between 70 – 84 (from –2 SD to < –1 SD)
- Moderate intellectual impairment will be an IQ between 55 – 69 (–3 SD to < –2 SD)
- Severe intellectual impairment will be an IQ below 55 (< –3 SD).

Attention & Executive Function

Preterm children are at increased risk of attention difficulties and executive dysfunction. Both are associated with diffuse white matter injury, which is the predominant form of brain injury in this population. Attention and executive function will be assessed using the following age standardised tests:

- Subtests from the Test of Everyday Attention for Children (TEACh) (Manly 1999) including Sky Search (selective attention), Score (sustained attention), Sky Search Dual Task (divided attention) and Creature Counting (shifting attention)
- The Rey Complex Figure (Rey 1993), which assesses spatial organisation and strategic decision-making
- The Fruit Stroop task (Archibald 1999), which assesses impulse control

In addition, parents and teachers will complete the Behavior Rating Inventory of Executive Function (BRIEF) (Gioia 2000), a questionnaire that assesses behavioural manifestations of inattention and executive function. The BRIEF provides eight theoretically and empirically derived clinical scales (Inhibit, Shift, Emotional Control, Initiate, Working Memory, Plan/Organize, Organization of Materials, Monitor) and scores are age and gender standardised. Internal consistency for the parent form of the BRIEF has been found to be high, ranging from 0.80 to 0.98 (Gioia 2000). Clinical validity has been supported with a variety of diagnostic groups.

Memory and Learning

The Rey Auditory Verbal Learning Test (RAVLT) (Rey 1964) will be administered to give multiple measures of verbal memory and learning, including proactive inhibition, retention, encoding versus retrieval, and subjective organization.

Visual-Perceptual Skills

Three subtests of the Test of Visual-Perceptual Skills, 3rd edition (TVPS-3), will be administered (Visual-spatial relations, Visual figure ground, and Visual closure) to assess visual-perceptual skills (Martin 2006).

Educational Progress

Educational progress will be assessed using the Wide Range Achievement Test (WRAT4) (Wilkinson 2005). Three subtests will be administered: word reading, spelling, and arithmetic. Scale scores from 70-84 will define mild impairment in these educational domains, while scale scores <70 will define major impairment.

Behavioural Problems

Children born preterm are at increased risk of attention-deficit hyperactivity disorder (ADHD). This will be assessed using a specific ADHD diagnostic questionnaire, namely, the Conners' (2000) ADHD/DSM-IV Scales (CADS). In addition, parent and teacher versions of the Strength & Difficulties Questionnaire (SDQ) will be administered (Goodman 1997). The SDQ is a well-validated questionnaire that assesses overall behaviour problems, emotional symptoms, hyperactivity/inattention, peer relationship problems, and prosocial behaviour.

APPENDIX 3: PHYSIOLOGICAL STUDIES

Ambulatory Blood pressure

Ambulatory blood pressure monitoring (ABPM) provides important information on circadian rhythm and blood pressure load. It also avoids the phenomenon of 'white coat hypertension', which is a common problem in children. ABPM is more sensitive for the detection of early hypertension and has greater predictive value for cardiovascular disease than casual BP measurement (O'Brien 2003). ABPM has been used successfully in large numbers of children and is generally well tolerated.

ABPM will be performed using the Spacelabs 90217 monitor and accompanying software. The cuff will be fitted to the non-dominant arm and cuff size will be selected according to the mid-arm circumference. Children will be asked to wear the monitor for a 24-hour period but remove it for washing or vigorous activity. One ABPM recording will be taken in the clinic on the day of assessment. The ABPM will be programmed to inflate every half hour from 07:00 to 24:00 and hourly from 24:00 to 07:00. Sleep and wake times will be recorded. A minimum of 14 daytime and 7 nighttime measurements will be required for acceptability (O'Brien 2000).

Reference ranges for casual blood pressure measurements are inappropriate for the interpretation of ABPM data (Diaz 2007). Therefore, ABPM results will be compared to a Central European reference population (Soergel 1997), which is the largest study of ABPM in healthy children and was also performed using Spacelabs monitors. This data has been updated using the LMS method for reference centile calculation (Cole 1992) to account for a non-Gaussian distribution (Wuhl 2002). LMS values will be determined from reference tables by linear interpolation for height and will be used to calculate ABPM parameter Z scores (systolic, diastolic and mean arterial pressure and heart rate). Blood pressure load will be determined for the 95th and 90th reference percentiles (Wuhl 2002). A normal nocturnal dip will be defined as a fall in mean daytime blood pressure of 10% or more during sleep.

Glucose Homeostasis

Children who assent to blood sampling will undergo a frequently sampled intravenous glucose tolerance test (FSIVGTT) using the shortened (90 minutes) method for children (Cutfield 1990) with insulin modification (Saad, Steil, Kades, 1997; Saad, Steil, Riad-Gabriel, 1997). This requires an overnight fast and insertion of a single intravenous cannula after application of local anaesthetic cream (EMLA). Heparinised saline will be dripped through the cannula to ensure patency and allow frequent blood sampling for insulin and glucose. This test has been successfully performed at the Liggins Institute in large numbers of children as young as 4 years of age, without apparent distress or adverse effect. The test will be abandoned if intravenous access is difficult or the child has persisting symptomatic hypoglycemia after the insulin bolus, although this is uncommon.

Glucose will be measured using a Hitachi autoanalyser and insulin will be measured using the IMX Abbott immunoassay, both of which are available at the Liggins Institute.

Glucose and insulin dynamics will be analysed via the Bergman minimal model using MINMOD Millennium (v.6) software (Boston 2003). This method is well validated and highly correlated with clamp techniques (Bergman 2005; Cutfield 1990; Saad, Steil, Kades, 1997). MINMOD assesses four different parameters:

- Insulin sensitivity index (S_I), which is the ability of insulin to accelerate net glucose disposal.
- Acute insulin response, which is the insulin released in the first 10 minutes after the glucose bolus.
- Glucose effectiveness (Sg), which is the ability of glucose to enhance its own disposal and suppress its production at basal insulin levels.
- Glucose disappearance coefficient (Kg), which is calculated from the slope of the natural log of glucose concentration between 10 and 19 minutes.

Bone Mass & Body Composition

Childhood and adolescence are critical periods for bone mineral accrual and achievement of optimal early bone mass is an important factor in preventing osteoporosis in adulthood (Horlick 2004; Specker 2005). Dual energy x-ray absorptiometry (DEXA) will be used to measure total body bone mass, also termed bone mineral content (BMC). DEXA is the most widely used densitometry technique in children (Gilsanz 1998; Kalkwarf 2007; Ward 2007) and whole body scans are preferred because of their greater precision compared to regional studies (Margulies 2005). Whole body scans are quick to perform in children

(usually less than 5 minutes) and the radiation dose is low and generally regarded as trivial (Njeh 1999; Sanchez 2005). Cortical bone composes 80% of the total skeletal mass, therefore, whole body DEXA reflects predominantly cortical bone mass and dimensions (Leonard 2004).

BMC (g) depends on both the size and density of bones. However, DEXA cannot determine true bone density as it measures only cross sectional bone area (BA, cm^2) and not bone volume. The increase in BMC that occurs throughout childhood is due mainly to changes in bone size as true volumetric bone density (vBMD, g/cm^3), as measured by quantitative computed tomography (QCT), is relatively constant until puberty (Specker 2005). Interpretation of paediatric DEXA is complicated by the fact that large differences can occur in body and bone size within and across different ages. Therefore, BMC values must be adjusted for size but the BMC/BA index, also known as the areal bone mineral density (aBMD, g/cm^2), provides only a crude adjustment with larger bones having an artificially inflated aBMD for a given vBMD (Specker 2005). Therefore, total body BMC (TBBMC) should be interpreted in relation to body size as well as BA (Horlick 2004; Molgaard 1997; Prentice 1994). Gender, ethnicity and pubertal status are also important factors affecting BMC, although pubertal status adds little to prediction of TBBMC if adjustment is made for other variables including body size (Horlick 2004). When compared to QCT, TBBMC-for-height provides a better estimate of bone strength than TBBMC-for-BA and TBBA-for-height is the best index of bone dimension (Leonard 2004).

Whole body DEXA will also be used to measure body composition including total body fat mass (TBFM, g), lean mass (TBLM, g), fat percentage (FM%), fat distribution (android: gynoid ratio), and fat mass index (FM/ height). DEXA body composition measurements show a close relationship to those obtained by the four-compartment method, although small differences in FM% occur with body size and between manufacturers (Sopher 2004).

DEXA results differ between manufacturer and machine. Therefore, a single type of machine will be used, the Lunar Prodigy, which is available at the Liggins (Encore v 8.1) and St George's Radiology in Christchurch (Encore v8.8). Both facilities have the paediatric software module.

Cortisol

Salivary cortisol measurements are widely used in paediatric research to assess hypopituitarity-pituitary-adrenal (HPA) axis function (Hanrahan 2006; Jessop 2007). They allow for determination of basal cortisol levels in a stress-free manner in the home environment but can also be used to assess stress responses as salivary cortisol levels peak within minutes of a plasma cortisol surge (Levine 2007; Vining 1983). Saliva and plasma cortisol concentrations are highly correlated (Aardal 1995; Gallagher 2006; Poll 2007; Vining 1983; Woodside 1991), especially when salivary cortisol is related to the unbound plasma cortisol fraction (Kirschbaum 1989). Salivary cortisol concentrations are approximately 70% that of unbound plasma cortisol (Rosmalen 2005) and are unaffected by salivary flow rate (Kirschbaum 1989).

Basal cortisol concentrations will be determined from saliva collected at home on days representing normal activity, such as a school day. Morning fasting samples will be compared to early evening ones (5pm) to allow assessment of the diurnal pattern. Children will be instructed to collect the morning sample as soon as possible after waking. Because of day-to-day variations in cortisol secretion, samples will be collected over 3 days and averaged. The caregiver will record the time of waking and sample collection. Saliva samples will not be collected within half an hour of eating or brushing teeth.

Saliva will be collected by passive drool into Salivette tubes and children will be asked to collect a minimum of 1ml per sample. Cotton swabs will not be used as we have shown that they achieve variable cortisol recovery (unpublished data), a finding that has also been confirmed by other laboratories (Groschl 2006; Strazdins 2005). Caregivers will be instructed to freeze the Salivettes immediately after collection. Once all 6 tubes have been collected they will be posted back to the study centre and then stored at -20°C. We have confirmed that cortisol is stable in saliva at room temperature for up to 5 days and that cortisol concentrations are unaffected by 2 freeze-thaw cycles (unpublished data). This has also been shown in other studies (Aardal 1995; Clements 1998; Groschl 2001).

Cortisol will be assayed by mass spectrometry using a technique that has been well validated in our laboratory. Mass spectrometry has the advantage of allowing simultaneous measurement of other hormones and we will also assay dehydroepiandrosterone (DHEA) to allow calculation of the cortisol / DHEA ratio. Abnormal cortisol / DHEA ratios have been shown to be have clinical predictive value for psychopathology (Goodyer 2001).

In the subgroup of children who agree to undergo a FSIVGTT, we will measure plasma cortisol concentrations at baseline (fasting) and also following the insulin bolus, which may provide further information about HPA axis function in relation to physiological stress.

Renal Function

Plasma creatinine will be measured in the baseline blood of those children undergoing FSIVGTT via the Liggins Hitachi autoanalyser. Creatinine clearance (ml/min/1.73m^2) will be estimated from height using the Schwartz (1976) equation:

$$eGFR = kL/Scr \ (ml/min/1.73m^2)$$

L = height in cm. Scr = creatinine concentration in mg/dl. The value of k is 0.47 for children when using the creatinase method of modern autoanalysers (Schwartz 2007). eGFR shows good correlation with creatinine clearance (r=0.935) (Schwartz 1976).

REFERENCES

Conners' Rating Scales-Revised Technical Manual. North Tonawanda, New York: Multi Health Systems; 2000.

Aardal E, Holm AC. Cortisol in saliva--reference ranges and relation to cortisol in serum. Eur J Clin Chem Clin Biochem. 1995;33(12):927-32.

Abbasi S, Hirsch D, Davis J, Tolosa J, Stouffer N, Debbs R, *et al.* Effect of single versus multiple courses of antenatal corticosteroids on maternal and neonatal outcome. Am J Obstet Gynecol. 2000;182(5):1243-9.

Aghajafari F, Murphy K, Matthews S, Ohlsson A, Amankwah K, Hannah M. Repeated doses of antenatal corticosteroids in animals: a systematic review. Am J Obstet Gynecol. 2002;186(4):843-9.

Aghajafari F, Murphy K, Ohlsson A, Amankwah K, Matthews S, Hannah ME. Multiple versus single courses of antenatal corticosteroids for preterm birth: a pilot study. J Obstet Gynaecol Can. 2002;24(4):321-9.

American Academy of Pediatrics. Eye examination in infants, children, and young adults by pediatricians. Pediatrics. 2003;111(4 Pt 1):902-7.

American Thoracic Society. Standardization of Spirometry, 1994 Update. Am J Respir Crit Care Med. 1995;152(3):1107-36.

Anand D, Stevenson CJ, West CR, Pharoah POD. Lung function and respiratory health in adolescents of very low birth weight. Arch Dis Child. 2003;88(2):135-8.

Archibald S, Kerns K. Identification and description of new tests of executive functioning in children. Child Neuropsych. 1999;5(2):115-29.

Ashwood PJ, Crowther CA, Willson KJ, Haslam RR, Kennaway DJ, Hiller JE, *et al.* Neonatal adrenal function after repeat dose prenatal corticosteroids: a randomized controlled trial. Am J Obstet Gynecol. 2006;194(3):861-7.

Banks BA, Cnaan A, Morgan MA, Parer JT, Merrill JD, Ballard PL, *et al.* Multiple courses of antenatal corticosteroids and outcome of premature neonates. North American Thyrotropin-Releasing Hormone Study Group. Am J Obstet Gynecol. 1999;181(3):709-17.

Barker DJP. The developmental origins of insulin resistance. Horm Res. 2005;64 Suppl 3:2-7.

Barker DJP, Bagby SP, Hanson MA. Mechanisms of disease: in utero programming in the pathogenesis of hypertension. Nat Clin Pract Nephrol. 2006;2(12):700-7.

Battin MR, Bevan C, Harding JE. Repeat doses of antenatal steroids and hypothalamic-pituitary-adrenal axis (HPA) function. Am J Obstet Gynecol. 2007;197(1):40.e1-6.

Bergman RN. Minimal model: perspective from 2005. Horm Res. 2005;64 Suppl 3:8-15.

Boggess KA, Bailit JL, Singer ME, Parisi VM, Mercer BM. Projected benefits of universal or scheduled antepartum corticosteroids to prevent neonatal morbidity: a decision analysis. Am J Obstet Gynecol. 2005;193(4):1415-23.

Boston RC, Stefanovski D, Moate PJ, Sumner AE, Watanabe RM, Bergman RN. MINMOD Millennium: a computer program to calculate glucose effectiveness and insulin sensitivity from the frequently sampled intravenous glucose tolerance test. Diabetes Technol Ther. 2003;5(6):1003-15.

Brocklehurst P, Gates S, McKenzie-McHarg K, Alfirevic Z, Chamberlain G. Are we prescribing multiple courses of antenatal corticosteroids? A survey of practice in the UK. Br J Obstet Gynaecol. 1999;106(9):977-9.

Callaghan W, MacDorman M, Rasmussen S, Qin C, Lackritz E. The Contribution of Preterm Birth to Infant Mortality Rates in the United States. Pediatrics. 2006;118(4):1566-73.

Cans C, Dolk H, Platt MJ, Colver A, Prasauskiene A, Krageloh-Mann I, *et al.* Recommendations from the SCPE collaborative group for defining and classifying cerebral palsy. Dev Med Child Neurol (Suppl). 2007;109:35-8.

Cleasby ME, Kelly PAT, Walker BR, Seckl JR. Programming of rat muscle and fat metabolism by in utero overexposure to glucocorticoids. Endocrinology. 2003;144(3):999-1007.

Clements AD, Parker CR. The relationship between salivary cortisol concentrations in frozen versus mailed samples. Psychoneuroendocrinology. 1998;23(6):613-6.

Cole TJ, Green PJ. Smoothing reference centile curves: the LMS method and penalized likelihood. Stat Med. 1992;11(10):1305-19.

Cole TJ, Freeman JV, Preece MA. Body mass index reference curves for the UK, 1990. Arch Dis Child. 1995;73(1):25-9.

Cole TJ, Freeman JV, Preece MA. British 1990 growth reference centiles for weight, height, body mass index and head circumference fitted by maximum penalized likelihood. Stat Med. 1998;17(4):407-29.

Cooper C, Fall C, Egger P, Hobbs R, Eastell R, Barker D. Growth in infancy and bone mass in later life. Ann Rheum Dis. 1997;56:17-21.

Craig E, Thompson J, Mitchell E. Socioeconomic status and preterm birth: New Zealand trends, 1980 to 1999. Arch Dis Child Fetal Neonatal Ed. 2002;86(3):F142-6.

Crowther CA, Haslam RR, Hiller JE, Doyle LW, Robinson JS. Neonatal respiratory distress syndrome after repeat exposure to antenatal corticosteroids: a randomised controlled trial. Lancet. 2006;367(9526):1913-9.

Crowther CA, Doyle LW, Haslam RR, Hiller JE, Harding JE, Robinson JS*, et al.* Outcomes at 2 years of age after repeat doses of antenatal corticosteroids. N Engl J Med. 2007;357(12):1179-89.

Crowther CA, McKinlay CJD, Middleton P, Harding JE. Repeat doses of prenatal corticosteroids for women at risk of preterm birth for improving neonatal health outcomes. Cochrane Database Sys Rev. 2011;Issue 6. Art. No. CD003935. DOI: 10.1002/14651858.CD003935.pub3.

Cutfield WS, Bergman RN, Menon RK, Sperling MA. The modified minimal model: application to measurement of insulin sensitivity in children. J Clin Endocrinol Metab. 1990;70(6):1644-50.

Dalziel SR, Liang A, Parag V, Rodgers A, Harding JE. Blood pressure at 6 years of age after prenatal exposure to betamethasone: follow-up results of a randomized, controlled trial. Pediatrics. 2004;114(3):e373-7.

Dalziel SR, Lim VK, Lambert A, McCarthy D, Parag V, Rodgers A*, et al.* Antenatal exposure to betamethasone: psychological functioning and health related quality of life 31 years after inclusion in randomised controlled trial. BMJ. 2005;331(7518):665.

Dalziel SR, Walker NK, Parag V, Mantell C, Rea HH, Rodgers A*, et al.* Cardiovascular risk factors after antenatal exposure to betamethasone: 30-year follow-up of a randomised controlled trial. Lancet. 2005;365(9474):1856-62.

Dalziel SR, Fenwick S, Cundy T, Parag V, Beck TJ, Rodgers A*, et al.* Peak bone mass after exposure to antenatal betamethasone and prematurity: follow-up of a randomized controlled trial. J Bone Miner Res. 2006;21(8):1175-86.

Dalziel SR, Rea HH, Walker NK, Parag V, Mantell C, Rodgers A*, et al.* Long term effects of antenatal betamethasone on lung function: 30 year follow up of a randomised controlled trial. Thorax. 2006;61(8):678-83.

Dalziel SR, Lim VK, Lambert A, McCarthy D, Parag V, Rodgers A*, et al.* Psychological functioning and health-related quality of life in adulthood after preterm birth. Dev Med Child Neurol. 2007;49(8):597-602.

Davis EP, Townsend EL, Gunnar MR, Georgieff MK, Guiang SF, Ciffuentes RF*, et al.* Effects of prenatal betamethasone exposure on regulation of stress physiology in healthy premature infants. Psychoneuroendocrinology. 2004;29(8):1028-36.

Davis EP, Townsend EL, Gunnar MR, Guiang SF, Lussky RC, Cifuentes RF*, et al.* Antenatal betamethasone treatment has a persisting influence on infant HPA axis regulation. J Perinatol. 2006;26(3):147-53.

Dessens AB, Haas HS, Koppe JG. Twenty-year follow-up of antenatal corticosteroid treatment. Pediatrics. 2000;105(6):E77.

Diaz LN, Garin EH. Comparison of ambulatory blood pressure and Task Force criteria to identify pediatric hypertension. Pediatr Nephrol. 2007;22(4):554-8.

Dodic M, May CN, Wintour EM, Coghlan JP. An early prenatal exposure to excess glucocorticoid leads to hypertensive offspring in sheep. Clin Sci (Colch). 1998;94(2):149-55.

Doyle LW, Ford GW, Rickards AL, Kelly EA, Davis NM, Callanan C, *et al.* Antenatal corticosteroids and outcome at 14 years of age in children with birth weight less than 1501 grams. Pediatrics. 2000;106(1):E2.

Doyle LW, Faber B, Callanan C, Morley R. Blood pressure in late adolescence and very low birth weight. Pediatrics. 2003;111(2):252-7.

Doyle LW, Victorian Infant Collaborative Study G. Respiratory function at age 8-9 years in extremely low birthweight/very preterm children born in Victoria in 1991-1992. Pediatr Pulmonol. 2006;41(6):570-6.

Dunlop SA, Archer MA, Quinlivan JA, Beazley LD, Newnham JP. Repeated prenatal corticosteroids delay myelination in the ovine central nervous system. J Matern Fetal Med. 1997;6(6):309-13.

Edwards CR, Benediktsson R, Lindsay RS, Seckl JR. Dysfunction of placental glucocorticoid barrier: link between fetal environment and adult hypertension? Lancet. 1993;341(8841):355-7.

Elimian A, Verma U, Visintainer P, Tejani N. Effectiveness of multidose antenatal steroids. Obstet Gynecol. 2000;95(1):34-6.

Eriksson JG, Forsen T, Tuomilehto J, Winter PD, Osmond C, Barker DJ. Catch-up growth in childhood and death from coronary heart disease: longitudinal study. BMJ. 1999;318(7181):427-31.

Esplin M, Fumsett M, Smith S. Multiple courses of antenatal steroids associated with a delay in long-term psychomotor development in children. Am J Obstet Gynecol. 2000;182:A27.

Fewtrell MS, Lucas A, Cole TJ, Wells JCK. Prematurity and reduced body fatness at 8-12 y of age. Am J Clin Nutr. 2004;80(2):436-40.

Ford LR, Willi SM, Hollis BW, Wright NM. Suppression and recovery of the neonatal hypothalamic-pituitary-adrenal axis after prolonged dexamethasone therapy. J Pediatr. 1997;131(5):722-6.

Fowden AL, Szemere J, Hughes P, Gilmour RS, Forhead AJ. The effects of cortisol on the growth rate of the sheep fetus during late gestation. J Endocrinol. 1996;151(1):97-105.

Freeman J, Cole T, Chinn S, Jones P, White E, Preece M. Cross sectional stature and weight reference curves for the UK, 1990. Arch Dis Child. 1995;73:17-24.

French NP, Hagan R, Evans SF, Godfrey M, Newnham JP. Repeated antenatal corticosteroids: size at birth and subsequent development. Am J Obstet Gynecol. 1999;180(1 Pt 1):114-21.

French NP, Hagan R, Evans SF, Mullan A, Newnham JP. Repeated antenatal corticosteroids: effects on cerebral palsy and childhood behavior. Am J Obstet Gynecol. 2004;190(3):588-95.

Galdes-Sebaldt M, Sheller JR, Grogaard J, Stahlman M. Prematurity is associated with abnormal airway function in childhood. Pediatr Pulmonol. 1989;7(4):259-64.

Gale CR, O'Callaghan FJ, Godfrey KM, Law CM, Martyn CN. Critical periods of brain growth and cognitive function in children. Brain. 2004;127(Pt 2):321-9.

Gallagher P, Leitch MM, Massey AE, McAllister-Williams RH, Young AH. Assessing cortisol and dehydroepiandrosterone (DHEA) in saliva: effects of collection method. J Psychopharmacol. 2006;20(5):643-9.

Gates S. Repeated antenatal steroid trial methodology. Am J Obstet Gynecol. 2007;197(4):437.

Gates S, Brocklehurst P. Decline in effectiveness of antenatal corticosteroids with time to birth: real or artefact? BMJ. 2007;335(7610):77-9.

Gilsanz V. Bone density in children: a review of the available techniques and indications. Eur J Radiol. 1998;26(2):177-82.

Gioia G, Isquith P, Guy S, Kenworthy L. BRIEF: Behavior Rating Inventory of Executive Function. Professional Manual. Odessa, FL: Psychological Assessment Resources; 2000.

Gluckman PD, Cutfield W, Hofman P, Hanson MA. The fetal, neonatal, and infant environments-the long-term consequences for disease risk. Early Hum Dev. 2005;81(1):51-9.

Gluckman PD, Hanson MA, Morton SMB, Pinal CS. Life-long echoes--a critical analysis of the developmental origins of adult disease model. Biol Neonate. 2005;87(2):127-39.

Gluckman PD, Hanson MA, Beedle AS. Non-genomic transgenerational inheritance of disease risk. Bioessays. 2007;29(2):145-54.

Godfrey K, Walker-Bone K, Robinson S, Taylor P, Shore S, Wheeler T, *et al.* Neonatal bone mass: influence of parental birthweight, maternal smoking, body composition, and activity during pregnancy. J Bone Miner Res. 2001;16(9):1694-703.

Godfrey KM, Lillycrop KA, Burdge GC, Gluckman PD, Hanson MA. Epigenetic mechanisms and the mismatch concept of the developmental origins of health and disease. Pediatr Res. 2007;61(5 Pt 2):5R-10R.

Goodman R. The Strengths and Difficulties Questionnaire. J Child Psych Psychiat. 1997;38:581-6.

Goodyer IM, Park RJ, Netherton CM, Herbert J. Possible role of cortisol and dehydroepiandrosterone in human development and psychopathology. Br J Psychiatry. 2001;179:243-9.

Groschl M, Wagner R, Rauh M, Dorr H-G. Stability of salivary steroids: the influences of storage, food and dental care. Steroids. 2001;66(10):737-41.

Groschl M, Rauh M. Influence of commercial collection devices for saliva on the reliability of salivary steroids analysis. Steroids. 2006;71(13-14):1097-100.

Guinn DA, Atkinson MW, Sullivan L, Lee M, MacGregor S, Parilla BV, *et al.* Single vs weekly courses of antenatal corticosteroids for women at risk of preterm delivery: a randomized controlled trial. JAMA. 2001;286(13):1581-7.

Hack M, Fanaroff AA. Outcomes of children of extremely low birthweight and gestational age in the 1990's. Early Hum Dev. 1999;53(3):193-218.

Hanrahan K, McCarthy AM, Kleiber C, Lutgendorf S, Tsalikian E. Strategies for salivary cortisol collection and analysis in research with children. Appl Nurs Res. 2006;19(2):95-101.

Harding JE, Pang J, Knight DB, Liggins GC. Do antenatal corticosteroids help in the setting of preterm rupture of membranes? Am J Obstet Gynecol. 2001;184(2):131-9.

Hasbargen U, Reber D, Versmold H, Schulze A. Growth and development of children to 4 years of age after repeated antenatal steroid administration. Eur J Pediatr. 2001;160(9):552-5.

Henderson S, Sugden D, Barnett A. Movement assessment battery for children-2. London: Harcourt Assessment; 2007.

Hibbert ME, Lannigan A, Landau LI, Phelan PD. Lung function values from a longitudinal study of healthy children and adolescents. Pediatr Pulmonol. 1989;7(2):101-9.

Hofman PL, Regan F, Jackson WE, Jefferies C, Knight DB, Robinson EM, *et al.* Premature birth and later insulin resistance. N Engl J Med. 2004;351(21):2179-86.

Horlick M, Wang J, Pierson RN, Jr., Thornton JC. Prediction models for evaluation of total-body bone mass with dual-energy X-ray absorptiometry among children and adolescents. Pediatrics. 2004;114(3):e337-45.

Huang WL, Beazley LD, Quinlivan JA, Evans SF, Newnham JP, Dunlop SA. Effect of corticosteroids on brain growth in fetal sheep. Obstet Gynecol. 1999;94(2):213-8.

Huang WL, Harper CG, Evans SF, Newnham JP, Dunlop SA. Repeated prenatal corticosteroid administration delays myelination of the corpus callosum in fetal sheep. Int J Dev Neurosci. 2001;19(4):415-25.

Ikegami M, Jobe A, Newnham J, Polk D, Willet K, Sly P. Repetitive prenatal glucocorticoids improve lung function and decrease growth in preterm lambs. Am J Respir Crit Care Med. 1997;156(1):178-84.

Irving R, Belton N, Elton R, Walker R. Adult cardiovascular risk factors in premature babies. Lancet. 2000;355(9221):2135-36.

Jessop DS, Turner-Cobb JM. Measurement and meaning of salivary cortisol: A focus on health and disease in children. Stress. 2007:1-14

Johns D, Pierce R. Pocket guide to spirometry. Syndey: McGraw-Hill; 2003.

Jongmans M, Mercuri E, Dubowitz L, Henderson S. Perceptual-motor diffculties and their concomitants in six-year-old children born prematurely. Human Movement Science. 1998;17:629-53.

Joseph K, Kramer M, Marcoux S, Ohlsson A, Wen S, Allen A, *et al.* Determinants of preterm birth rates in canada from 1981 through 1983 and from 1992 through 1994. New Engl J Med. 1998;339(20):1434-39.

Kalkwarf HJ, Zemel BS, Gilsanz V, Lappe JM, Horlick M, Oberfield S, *et al.* The bone mineral density in childhood study: bone mineral content and density according to age, sex, and race. J Clin Endocrinol Metab. 2007;92(6):2087-99.

Kirschbaum C, Hellhammer DH. Salivary cortisol in psychobiological research: an overview. Neuropsychobiology. 1989;22(3):150-69.

Kumar P, Seshadri R, Grobman WA. Neurodevelopmental outcome of very low birth weight infants after multiple courses of antenatal corticosteroids. J Soc Gynecol Investig. 2004;11(7):483-7.

Langley-Evans SC. Intrauterine programming of hypertension by glucocorticoids. Life Sci. 1997;60(15):1213-21.

Leon DA, Johansson M, Rasmussen F. Gestational age and growth rate of fetal mass are inversely associated with systolic blood pressure in young adults: an epidemiologic study of 165,136 Swedish men aged 18 years. Am J Epidemiol. 2000;152(7):597-604.

Leonard MB, Shults J, Elliott DM, Stallings VA, Zemel BS. Interpretation of whole body dual energy X-ray absorptiometry measures in children: comparison with peripheral quantitative computed tomography. Bone. 2004;34(6):1044-52.

Levine A, Zagoory-Sharon O, Feldman R, Lewis JG, Weller A. Measuring cortisol in human psychobiological studies. Physiol Behav. 2007;90(1):43-53.

Levitt NS, Lindsay RS, Holmes MC, Seckl JR. Dexamethasone in the last week of pregnancy attenuates hippocampal glucocorticoid receptor gene expression and elevates blood pressure in the adult offspring in the rat. Neuroendocrinology. 1996;64(6):412-8.

Liggins GC, Howie RN. A controlled trial of antepartum glucocorticoid treatment for prevention of the respiratory distress syndrome in premature infants. Pediatrics. 1972;50(4):515-25.

Lucas JS, Inskip HM, Godfrey KM, Foreman CT, Warner JO, Gregson RK, *et al.* Small size at birth and greater postnatal weight gain: relationships to diminished infant lung function. Am J Respir Crit Care Med. 2004;170(5):534-40.

MacArthur B, Howie R, Dezoete J, Elkins J. School progress and cognitive development of 6-year old children whose mothers were treated antenatally with betamethasone. Pediatrics. 1982;70(1):99-105.

Manly T, Robertson I, Anderson V, Nimmo-Smith I. The Test of Everyday Attention for Children. Suffolk, UK: Thames Valley Test Company; 1999.

Margulies L, Horlick M, Thornton JC, Wang J, Ioannidou E, Heymsfield SB. Reproducibility of pediatric whole body bone and body composition measures by dual-energy X-ray absorptiometry using the GE Lunar Prodigy. J Clin Densitom. 2005;8(3):298-304.

Marshall WA, Tanner JM. Variations in the pattern of pubertal changes in girls. Arch Dis Child. 1969;44(235):291-303.

Marshall WA, Tanner JM. Variations in the pattern of pubertal changes in boys. Arch Dis Child. 1970;45(239):13-23.

Martin BC, Warram JH, Krolewski AS, Bergman RN, Soeldner JS, Kahn CR. Role of glucose and insulin resistance in development of type 2 diabetes mellitus: results of a 25-year follow-up study. Lancet. 1992;340(8825):925-9.

Martin N. Test of Visual-Perceptual Skills (no-motor). 3rd ed: Academic Therapy Publications; 2006.

McEvoy C, Bowling S, Williamson K, Collins J, Tolaymat L, Maher J. Timing of antenatal corticosteroids and neonatal pulmonary mechanics. Am J Obstet Gynecol. 2000;183(4):895-9.

McEvoy C, Bowling S, Williamson K, Lozano D, Tolaymat L, Izquierdo L*, et al.* The effect of a single remote course versus weekly courses of antenatal corticosteroids on functional residual capacity in preterm infants: a randomized trial. Pediatrics. 2002;110(2 Pt 1):280-4.

McLaughlin KJ, Crowther CA, Vigneswaran P, Hancock E, Willson K. Who remains undelivered more than seven days after a single course of prenatal corticosteroids and gives birth at less than 34 weeks? Aust N Z J Obstet Gynaecol. 2002;42(4):353-7.

McLaughlin KJ, Crowther CA, Walker N, Harding JE. Effects of a single course of corticosteroids given more than 7 days before birth: a systematic review. Aust NZ J Obstet Gynaecol. 2003;43(2):101-6.

Mildenhall LFJ, Battin MR, Morton SMB, Bevan C, Kuschel CA, Harding JE. Exposure to repeat doses of antenatal glucocorticoids is associated with altered cardiovascular status after birth. Arch Dis Child Fetal Neonatal Ed. 2006;91(1):F56-60.

Modi N, Lewis H, Al-Naqeeb N, Ajayi-Obe M, Dore CJ, Rutherford M. The effects of repeated antenatal glucocorticoid therapy on the developing brain. Pediatr Res. 2001;50(5):581-5.

Molgaard C, Thomsen BL, Prentice A, Cole TJ, Michaelsen KF. Whole body bone mineral content in healthy children and adolescents. Arch Dis Child. 1997;76(1):9-15.

Moss TJM, Doherty DA, Nitsos I, Sloboda DM, Harding R, Newnham JP. Effects into adulthood of single or repeated antenatal corticosteroids in sheep. Am J Obstet Gynecol. 2005;192(1):146-52.

Murphy K. MULTIPLE COURSES OF ANTENATAL CORTICOSTEROIDS FOR PRETERM BIRTH: MACS COLLABORATIVE GROUP (Abstract). Am J Obstet Gynaecol. 2007;197(6):Suppl 1; S2.

National Institutes of Health. Effect of corticosteroids for fetal maturation on perinatal outcomes. Consensus Statement. 1994;12(2):1-24.

National Institutes of Health. Antenatal corticosteroids revisited: repeat courses. NIH Consensus Statement. 2000;17(2):1-18.

New Zealand Health Information Service. Maternity and newborn information. http://www.moh.govt.nz/moh.nsf/indexns/stats; 2008.

Ng P, Wong G, Lam C, Lee C, Fok T, Wong M, *et al.* Pituitary-adrenal suppression and recovery in preterm very low birth weight infants after dexamethasone treatment for bronchopulmonary dysplasia. J Clin Endocrinol Metab. 1997;82(8):2429-32.

Njeh CF, Fuerst T, Hans D, Blake GM, Genant HK. Radiation exposure in bone mineral density assessment. Appl Radiat Isot. 1999;50(1):215-36.

O'Brien E. Replacing the mercury sphygmomanometer. Requires clinicians to demand better automated devices. BMJ. 2000;320(7238):815-6.

O'Brien E, Asmar R, Beilin L, Imai Y, Mallion J-M, Mancia G, *et al.* European Society of Hypertension recommendations for conventional, ambulatory and home blood pressure measurement. J Hypertens. 2003;21(5):821-48.

Owen D, Banjanin S, Gidrewicz D, McCabe L, Matthews SG. Central regulation of the hypothalamic-pituitary-adrenal axis during fetal development in the Guinea-pig. J Neuroendocrinol. 2005;17(4):220-6.

Palisano R, Rosenbaum P, Walter S, Russell D, Wood E, Galuppi B. Development and reliability of a system to classify gross motor function in children with cerebral palsy. Dev Med Child Neurol. 1997;39(4):214-23.

Pe´rez-Padilla R, Va´zquez-Garcı´a J, Ma´rquez M, Jardim J, Pertuze J, Lisboa C, *et al.* The long-term stability of portable spirometers used in a multinational study of the prevalence of Chronic Obstructive Pulmonary Disease. Respir Care. 2006;51(10):1167-71.

Peltoniemi O, Kari M, Tammela O, Lehtonen L, Marttila R, Halmesmäki E, *et al.* Randomized trial of a single repeat dose of prenatal betamethasone treatment in imminent preterm birth. Pediatrics. 2007;119(2):290-8.

Pickering TG, Hall JE, Appel LJ, Falkner BE, Graves JW, Hill MN, *et al.* Recommendations for blood pressure measurement in humans: an AHA scientific statement from the Council on High Blood Pressure Research Professional and Public Education Subcommittee. J Clin Hypertens (Greenwich). 2005;7(2):102-9.

Pike K, Hanson M, Godfrey K. Developmental mismatch: consequences for later cardiorespiratory health. British J Obstet Gynaecol. 2008;115:149-57.

Poll E-M, Kreitschmann-Andermahr I, Langejuergen Y, Stanzel S, Gilsbach JM, Gressner A, *et al.* Saliva collection method affects predictability of serum cortisol. Clin Chim Acta. 2007;382(1-2):15-9.

Prentice A, Parsons TJ, Cole TJ. Uncritical use of bone mineral density in absorptiometry may lead to size-related artifacts in the identification of bone mineral determinants. Am J Clin Nutr. 1994;60(6):837-42.

Quinlivan JA, Archer MA, Dunlop SA, Evans SF, Beazley LD, Newnham JP. Fetal growth retardation, particularly within lymphoid organs, following repeated maternal injections of betamethasone in sheep. J Obstet Gynaecol Res. 1998;24(3):173-82.

Quinlivan JA, Evans SF, Dunlop SA, Beazley LD, Newnham JP. Use of corticosteroids by Australian obstetricians--a survey of clinical practice. Aust N Z J Obstet Gynaecol. 1998;38(1):1-7.

Regan FM, Cutfield WS, Jefferies C, Robinson E, Hofman PL. The impact of early nutrition in premature infants on later childhood insulin sensitivity and growth. Pediatrics. 2006;118(5):1943-9.

Rey A. L'examen clinique en psychologie. Paris: Presses Universitaires de France; 1964.

Rey A. Psychological examination of traumatic encephalopathy (translation). The Clinical Neuropsychologist. 1993;7:3-21.

Rickards A, Kitchen W, Doyle L, Kelly E. Correction of developmental and intelligence test scores for premature birth. 1989;25:127-9.

Roberts D, Dalziel S. Antenatal corticosteroids for accelerating fetal lung maturation for women at risk of preterm birth. Cochrane Database Syst Rev. 2006;(3):Art. No.: CD004454. DOI: 10.1002/14651858.CD004454.pub2.

Rose SA, Feldman JF, Jankowski JJ. The building blocks of cognition. J Pediatr. 2003;143(4 Suppl):S54-61.

Rosmalen JGM, Oldehinkel AJ, Ormel J, de Winter AF, Buitelaar JK, Verhulst FC. Determinants of salivary cortisol levels in 10-12 year old children: a population-based study of individual differences. Psychoneuroendocrinology. 2005;30(5):483-95.

Rosner B, Prineas R, Loggie J, Daniels S. Blood pressure nomograms for children and adolescents, by height, sex, and age, in the United States. J Pediatr. 1993;123(6):871-86.

Saad MF, Steil GM, Kades WW, Ayad MF, Elsewafy WA, Boyadjian R*, et al.* Differences between the tolbutamide-boosted and the insulin-modified minimal model protocols. Diabetes. 1997;46(7):1167-71.

Saad MF, Steil GM, Riad-Gabriel M, Khan A, Sharma A, Boyadjian R*, et al.* Method of insulin administration has no effect on insulin sensitivity estimates from the insulin-modified minimal model protocol. Diabetes. 1997;46(12):2044-8.

Saigal S, Feeny D, Furlong W, Rosenbaum P, Burrows E, Torrance G. Comparison of the health-related quality of life of extremely low birth weight children and a reference group of children at age eight years. J Pediatr. 1994;125(3):418-25.

Sanchez MM, Gilsanz V. Pediatric DXA bone measurements. Pediatr Endocrinol Rev. 2005;2 Suppl 3:337-41.

Schmand B, Neuvel J, Smolders-de Haas H, Hoeks J, Treffers PE, Koppe JG. Psychological development of children who were treated antenatally with corticosteroids to prevent respiratory distress syndrome. Pediatrics. 1990;86(1):58-64.

Schwartz GJ, Haycock GB, Edelmann CM, Jr., Spitzer A. A simple estimate of glomerular filtration rate in children derived from body length and plasma creatinine. Pediatrics. 1976;58(2):259-63.

Schwartz GJ, Furth SL. Glomerular filtration rate measurement and estimation in chronic kidney disease. Pediatr Nephrol. 2007;22(11):1839-48.

Seckl JR. Glucocorticoid programming of the fetus; adult phenotypes and molecular mechanisms. Mol Cell Endocrinol. 2001;185(1-2):61-71.

Sewell J, Oberklaid F, Prior M, Sanson A, Kyrios M. Temperament in Australian toddlers. Aust Paediatr J. 1988;24(6):343-5.

Shaheen SO, Newson RB, Henderson AJ, Emmett PM, Sherriff A, Cooke M*, et al.* Umbilical cord trace elements and minerals and risk of early childhood wheezing and eczema. Eur Respir J. 2004;24(2):292-7.

Shinwell ES, Karplus M, Reich D, Weintraub Z, Blazer S, Bader D*, et al.* Early postnatal dexamethasone treatment and increased incidence of cerebral palsy. Arch Dis Child Fetal Neonatal Ed. 2000;83(3):F177-81.

Sloboda DM, Moss T, Gurrin L, Newnham JP, Challis J. The effect of prenatal betamethasone administration on postnatal ovine hypothalamic-pituitary-adrenal function. J Endocrinol. 2002;172(1):71-81.

Sloboda DM, Challis JRG, Moss TJM, Newnham JP. Synthetic glucocorticoids: antenatal administration and long-term implications. Curr Pharm Des. 2005;11(11):1459-72.

Sloboda DM, Moss T, Li S, Doherty D, Nitsos I, Challis JRG*, et al.* Prenatal betamethasone exposure results in pituitary-adrenal hyporesponsiveness in adult sheep. Am J Physiol Endocrinol Metab. 2007;292(1):E61-70.

Soergel M, Kirschstein M, Busch C, Danne T, Gellermann J, Holl R*, et al.* Oscillometric twenty-four-hour ambulatory blood pressure values in healthy children and adolescents: a multicenter trial including 1141 subjects. J Pediatr. 1997;130(2):178-84.

Sopher AB, Thornton JC, Wang J, Pierson RN, Jr., Heymsfield SB, Horlick M. Measurement of percentage of body fat in 411 children and adolescents: a comparison of dual-energy X-ray absorptiometry with a four-compartment model. Pediatrics. 2004;113(5):1285-90.

Specker BL, Schoenau E. Quantitative bone analysis in children: current methods and recommendations. J Pediatr. 2005;146(6):726-31.

Spinillo A, Viazzo F, Colleoni R, Chiara A, Maria Cerbo R, Fazzi E. Two-year infant neurodevelopmental outcome after single or multiple antenatal courses of corticosteroids to prevent complications of prematurity. Am J Obstet Gynecol. 2004;191(1):217-24.

Stewart JD, Sienko AE, Gonzalez CL, Christensen HD, Rayburn WF. Placebo-controlled comparison between a single dose and a multidose of betamethasone in accelerating lung maturation of mice offspring. Am J Obstet Gynecol 1998;179(5):1241-47.

Stick S. Pediatric origins of adult lung disease. 1. The contribution of airway development to paediatric and adult lung disease. Thorax. 2000;55(7):587-94.

Strazdins L, Meyerkort S, Brent V, D'Souza RM, Broom DH, Kyd JM. Impact of saliva collection methods on sIgA and cortisol assays and acceptability to participants. J Immunol Methods. 2005;307(1-2):167-71.

Stutchfield P, Whitaker R, Russell I. Antenatal betamethasone and incidence of neonatal respiratory distress after elective caesarean section: pragmatic randomised trial. BMJ. 2005;331(7518):662.

Swolin-Eide D, Dahlgren J, Nilsson C, Albertsson Wikland K, Holmang A, Ohlsson C. Affected skeletal growth but normal bone mineralization in rat offspring after prenatal dexamethasone exposure. J Endocrinol. 2002;174(3):411-8.

Takahashi R, Yamada M, Takahashi T, Ito T, Nakae S, Kobayashi Y*, et al.* Risk factors for cerebral palsy in preterm infants. Early Hum Dev. 2005;81(6):545-53.

Thompson C, Syddall H, Rodin I, Osmond C, Barker D. Birth weight and the risk of depressive disorder in late life. Br J Psychiatry. 2001;179:450-5.

Thompson DK, Warfield SK, Carlin JB, Pavlovic M, Wang HX, Bear M*, et al.* Perinatal risk factors altering regional brain structure in the preterm infant. Brain. 2007;130(Pt 3):667-77.

Thorp JA, Jones AM, Hunt C, Clark R. The effect of multidose antenatal betamethasone on maternal and infant outcomes. Am J Obstet Gynecol. 2001;184(2):196-202.

Tschanz SA, Damke BM, Burri PH. Influence of postnatally administered glucocorticoids on rat lung growth. Biol Neonate. 1995;68(4):229-45.

Tucker J, McGuire W. Epidemiology of preterm birth. BMJ. 2004;329:675-78.

Uthaya S, Thomas EL, Hamilton G, Dore CJ, Bell J, Modi N. Altered adiposity after extremely preterm birth. Pediatr Res. 2005;57(2):211-5.

Vining RF, McGinley RA, Maksvytis JJ, Ho KY. Salivary cortisol: a better measure of adrenal cortical function than serum cortisol. Ann Clin Biochem. 1983;20(Pt 6):329-35.

Walters J, Wood-baker R, Walls J, Johns D. Stability of the EasyOne ultrasonic spirometer for use in general practice. Respirology. 2006;11:306-10.

Wapner RJ, Sorokin Y, Thom EA, Johnson F, Dudley DJ, Spong CY*, et al.* Single versus weekly courses of antenatal corticosteroids: evaluation of safety and efficacy. Am J Obstet Gynecol. 2006;195(3):633-42.

Wapner RJ, Sorokin Y, Mele L, Johnson F, Dudley DJ, Spong CY*, et al.* Long-term outcomes after repeat doses of antenatal corticosteroids. N Engl J Med. 2007;357(12):1190-8.

Ward KA, Ashby RL, Roberts SA, Adams JE, Zulf Mughal M. UK reference data for the Hologic QDR Discovery dual-energy x ray absorptiometry scanner in healthy children and young adults aged 6-17 years. Arch Dis Child. 2007;92(1):53-9.

Waters E, Salmon L, Wake M, Hesketh K, Wright M. The Child Health Questionnaire in Australia: reliability, validity and population means. Aust N Z J Public Health. 2000;24(2):207-10.

Wechsler D. The Wechsler Abbreviated Scale of Intelligence. San Antonio: The Psychological Corporation; 1999.

Welberg LA, Seckl JR. Prenatal stress, glucocorticoids and the programming of the brain. J Neuroendocrinol. 2001;13(2):113-28.

Wilkinson G, Robertson G. Wide Range Achievement Test. 4th ed. Wilmington, Delaware; 2005.

Willet KE, Jobe AH, Ikegami M, Kovar J, Sly PD. Lung morphometry after repetitive antenatal glucocorticoid treatment in preterm sheep. Am J Respir Crit Care Med. 2001;163(6):1437-43.

Woodside DB, Winter K, Fisman S. Salivary cortisol in children: correlations with serum values and effect of psychotropic drug administration. Can J Psychiatry. 1991;36(10):746-8.

Wuhl E, Witte K, Soergel M, Mehls O, Schaefer F, German Working Group on Pediatric Hypertension. Distribution of 24-h ambulatory blood pressure in children: normalized reference values and role of body dimensions. J Hypertens. 2002;20(10):1995-2007.
