# Supplementary material for: Association of Fetal Growth Restriction With Neurocognitive Function After Repeated Antenatal Betamethasone Treatment vs Placebo: Secondary Analysis of the ACTORDS Randomized Clinical Trial
Source: JAMA Netw Open. 2019 Feb 1;2(2):e187636. doi: 10.1001/jamanetworkopen.2018.7636 (PMC6484607; doi:10.1001/jamanetworkopen.2018.7636)
Supplement: Supplement 2. — eTable. Primary and Secondary Neurocognitive Outcomes at Midchildhood of Children With and Without Fetal Growth Restriction (FGR) [file jamanetwopen-2-e187636-s002.pdf]

Supplementary Online Content

Cartwright R, Crowther CA, Anderson PJ, Harding JE, Doyle LW, McKinlay CJD. Association of fetal growth restriction and neurocognitive function after repeated antenatal betamethasone treatment vs placebo: secondary analysis of the ACTORDS randomized clinical trial. *JAMA Netw Open*. 2019;2(2):e187636. doi:10.1001/jamanetworkopen.2018.7636

eTable. Primary and Secondary Neurocognitive Outcomes at Midchildhood of Children With and Without Fetal Growth Restriction (FGR)

This supplementary material has been provided by the authors to give readers additional information about their work.

© 2018 Cartwright R et al. *JAMA Network Open.*

eTable. Primary and Secondary Neurocognitive Outcomes at Midchildhood of Children With and Without Fetal Growth Restriction (FGR)

Outcome	FGR	No. of Participants	Non-FGR	No. of Participants	Treatment effect: OR or MD (95% CI)	*P* Value
Survival free of any disability†	199 (73.7%)	270	550 (79.4%)	693	0.9 (0.9 to 1.0)	.10
Death or moderate/severe disability†	41 (15.2%)	270	65 (9.4%)	693	1.6 (1.1 to 2.4)	.02
Cognition						
Full scale IQ	97.4 (15.3)	241	100.7 (16.2)	648	-3.3 (-5.8 to -0.8)	.008
Full scale IQ <85	35 (14.5%)	241	81 (12.5%)	648	1.2 (0.8 to 1.7)	.44
Motor						
Cerebral palsy	11 (4.2%)	261	28 (3.9%)	726	1.1 (0.6 to 2.2)	.78
Low movement ABC Total Score (<15th centile)	79 (33.3%)	237	144 (22.9%)	630	1.5 (1.2 to 1.8)	.002
Attention: TEA-Ch						
Selective – Sky Search	9.0 (3.3)	233	9.2 (3.2)	621	-0.2 (-0.7 to 0.3)	.44
Sustained – Score!	8.9 (3.7)	226	8.7 (3.5)	604	0.2 (-0.4 to 0.8)	.51
Shifting – Creature Counting	9.0 (3.7)	194	9.8 (3.6)	553	-0.8 (-1.4 to -0.1)	.02
Divided – Sky Search Dual Task	56.6 (28.7)	216	60.3 (29.3)	582	-3.7 (-8.3 to 0.9)	.12
Executive Function						
Rey Complex Figure Accuracy Score	14.4 (7.3)	230	15.8 (7.9)	618	-1.5 (-2.7 to -0.3)	.02
Rey Complex Figure Organisation Score	3.6 (1.2)	230	3.7 (1.2)	615	-0.1 (-0.3 to 0.0)	.14
Fruit Stroop, number correct (trial 4)	19.6 (8.1)	225	20.4 (8.5)	604	-0.8 (-2.1 to 0.6)	.26
Academic skills						
Reading	97.0 (16.3)	228	99.9 (17.5)	630	-2.9 (-5.7 to 0.0)	.05
Spelling	98.5 (15.2)	226	100.8 (16.5)	628	-2.3 (-5.0 to 0.4)	.10
Mathematics	95.0 (15.5)	227	97.3 (16.0)	626	-2.3 (-4.9 to 0.3)	.08
Parental rating of behaviour						
SDQ Total Difficulties Score	10.9 (6.2)	229	10.8 (6.8)	636	0.1 (-0.9 to 1.2)	.81
BRIEF Global Executive Composite T-Score	52.0 (12.4)	226	52.3 (12.9)	631	-0.3 (-2.3 to 1.7)	.78
CADS ADHD Index T-Score	51.3 (6.5)	231	51.3 (7.0)	636	0.1 (-1.0 to 1.1)	.92

Data are percent (number) and mean (standard deviation). OR, odds ratio; MD, mean difference. IQ, intelligence quotient; TEA-Ch, Test of Everyday Attention for Children.

† Disability defined as any of cerebral palsy, blindness or deafness, or IQ <85; moderate/severe disability defined as deafness, moderate/severe cerebral palsy, or IQ<70. Subgroups compared using a generalised linear model. Adjusted for adjusted for clustering of children from multiple pregnancy.

© 2018 Cartwright R et al. *JAMA Network Open.*
